# Transmembrane tumor necrosis factor alpha attenuates pressure-overload cardiac hypertrophy via tumor necrosis factor receptor 2

**DOI:** 10.1371/journal.pbio.3000967

**Published:** 2020-12-03

**Authors:** Kun Miao, Ling Zhou, Hongping Ba, Chenxi Li, Haiyan Gu, Bingjiao Yin, Jing Wang, Xiang-ping Yang, Zhuoya Li, Dao Wen Wang

**Affiliations:** 1 Division of Cardiology, Department of Internal Medicine and Department of Hubei Key Laboratory of Genetics and Molecular Mechanisms of Cardiologic Disorders, Tongji Hospital, Tongji Medical College, Huazhong University of Science and Technology, Wuhan, Hubei, China; 2 Department of Immunology, School of Basic Medicine, Tongji Medical College, Huazhong University of Science and Technology, Wuhan, Hubei, China; University of Pittsburgh, UNITED STATES

## Abstract

Tumor necrosis factor-alpha (TNF-α) plays an important pathogenic role in cardiac hypertrophy and heart failure (HF); however, anti-TNF is paradoxically negative in clinical trials and even worsens HF, indicating a possible protective role of TNF-α in HF. TNF-α exists in transmembrane (tmTNF-α) and soluble (sTNF-α) forms. Herein, we found that TNF receptor 1 (TNFR1) knockout (KO) or knockdown (KD) by short hairpin RNA or small interfering RNA (siRNA) significantly alleviated cardiac hypertrophy, heart dysfunction, fibrosis, and inflammation with increased tmTNF-α expression, whereas TNFR2 KO or KD exacerbated the pathological phenomena with increased sTNF-α secretion in transverse aortic constriction (TAC)- and isoproterenol (ISO)-induced cardiac hypertrophy in vivo and in vitro, respectively, indicating the beneficial effects of TNFR2 associated with tmTNF-α. Suppressing TNF-α converting enzyme by TNF-α Protease Inhibitor-1 (TAPI-1) to increase endogenous tmTNF-α expression significantly alleviated TAC-induced cardiac hypertrophy. Importantly, direct addition of exogenous tmTNF-α into cardiomyocytes in vitro significantly reduced ISO-induced cardiac hypertrophy and transcription of the pro-inflammatory cytokines and induced proliferation. The beneficial effects of tmTNF-α were completely blocked by TNFR2 KD in H9C2 cells and TNFR2 KO in primary myocardial cells. Furthermore, we demonstrated that tmTNF-α displayed antihypertrophic and anti-inflammatory effects by activating the AKT pathway and inhibiting the nuclear factor (NF)-κB pathway via TNFR2. Our data suggest that tmTNF-α exerts cardioprotective effects via TNFR2. Specific targeting of tmTNF-α processing, rather than anti-TNF therapy, may be more useful for the treatment of hypertrophy and HF.

## Abstract 2

In contrast to detrimental effects of soluble tumor necrosis factor-alpha (TNF-α) via TNFR1, this study shows that transmembrane TNF-α protects the heart by suppressing pressure overload-induced cardiac hypertrophy and inflammation via TNFR2. Targeting tmTNF-α processing may be more useful than TNF-antagonist for treatment of hypertrophy and heart failure.

## Introduction

Serum levels of tumor necrosis factor-alpha (TNF-α) are elevated in patients with heart failure (HF) [1] and are an independent predictor of poor prognosis for the disease [2]. TNF-α inhibits cardiac contractility [3]; provokes myocardial hypertrophy [4], ventricular remodeling, and cardiac fibrosis; and induces cardiomyocyte apoptosis [5]. Chronic overexpression of TNF-α in the heart leads to dilated cardiomyopathy and increases mortality rates in mice [6]. To ameliorate the progression of HF caused by TNF-α, TNF-α antagonism has been used. Although anti-TNF therapies have improved cardiac function in several experimental animal models [7], large-scale, randomized, placebo-controlled clinical trials of TNF-α antagonists for the treatment of HF were abandoned early due to failed improvement of clinical status and mortality [8,9], and in some studies, the treatment even deteriorated HF [10,11]. These data suggest that TNF-α may play both protective and pathogenic roles in HF.

TNF-α exists as 2 bioactive forms, namely a 26-kDa transmembrane TNF-α (tmTNF-α) and a 17-kDa soluble TNF-α (sTNF-α). The latter represents the extracellular domain of tmTNF-α and can be released from the membrane-bound molecule at the cell surface through the cleavage by a disintegrin and metalloprotease 17 (also called TNF-α-converting enzyme [TACE]). Both forms of TNF-α are bioactive and display distinct functions [12,13]. TACE is up-regulated in human cardiomyopathy [14]. Additionally, transgenic mice with cardiac-specific overexpression of noncleavable tmTNF-α or sTNF-α exhibit disparate cardiac phenotypes; tmTNF-α favors concentric hypertrophy, whereas sTNF-α favors a dilated cardiac phenotype [15,16].

Both forms of TNF-α bind 2 TNF receptors (TNFR1 and TNFR2) with different affinities [17]. The effects of ligation with these 2 TNFRs vary in different cardiovascular diseases, such as HF [18], atherosclerosis [19], and ischemic injury [20,21]. TNFR1 mediates almost all known biological effects of sTNF-α in the heart, i.e., inducing hypertrophy, mediating the apoptosis of neonatal cardiomyocytes, and stimulating fibroblast proliferation and collagen synthesis. The function of TNFR2 is not well defined in comparison with that of TNFR1. Recent studies have suggested that TNFR2 exhibits cardioprotective effects against the progression of HF [18] and ischemic injury [22]. However, which ligand mediates the cardioprotective effect of TNFR2 is unclear.

tmTNF-α is the primary ligand of TNFR2 [17], and our previous study showed that tmTNF-α exerts anti-inflammatory and insulin-sensitizing effects in adipocytes [13]. Therefore, we hypothesized that tmTNF-α may have protective effects via TNFR2 in cardiac hypertrophy and HF. To test our hypothesis, we used wild-type (WT) and different TNFR knockout (KO) mice in a transverse aortic constriction (TAC)-induced pressure-overload hypertrophy model and isoproterenol (ISO)-induced cardiac hypertrophy in vitro to evaluate the functions of tmTNF-α. Our data provide important insights into the protective role of tmTNF-α in pressure-overload hypertrophy and use of selective blockage of tmTNF-α shedding as a potential therapeutic strategy in the treatment of hypertrophy and HF.

## Results

### Increased tmTNF-α expression is involved in the protective effects of TNFR2 on TAC-induced cardiac hypertrophy

**Fig 1:**
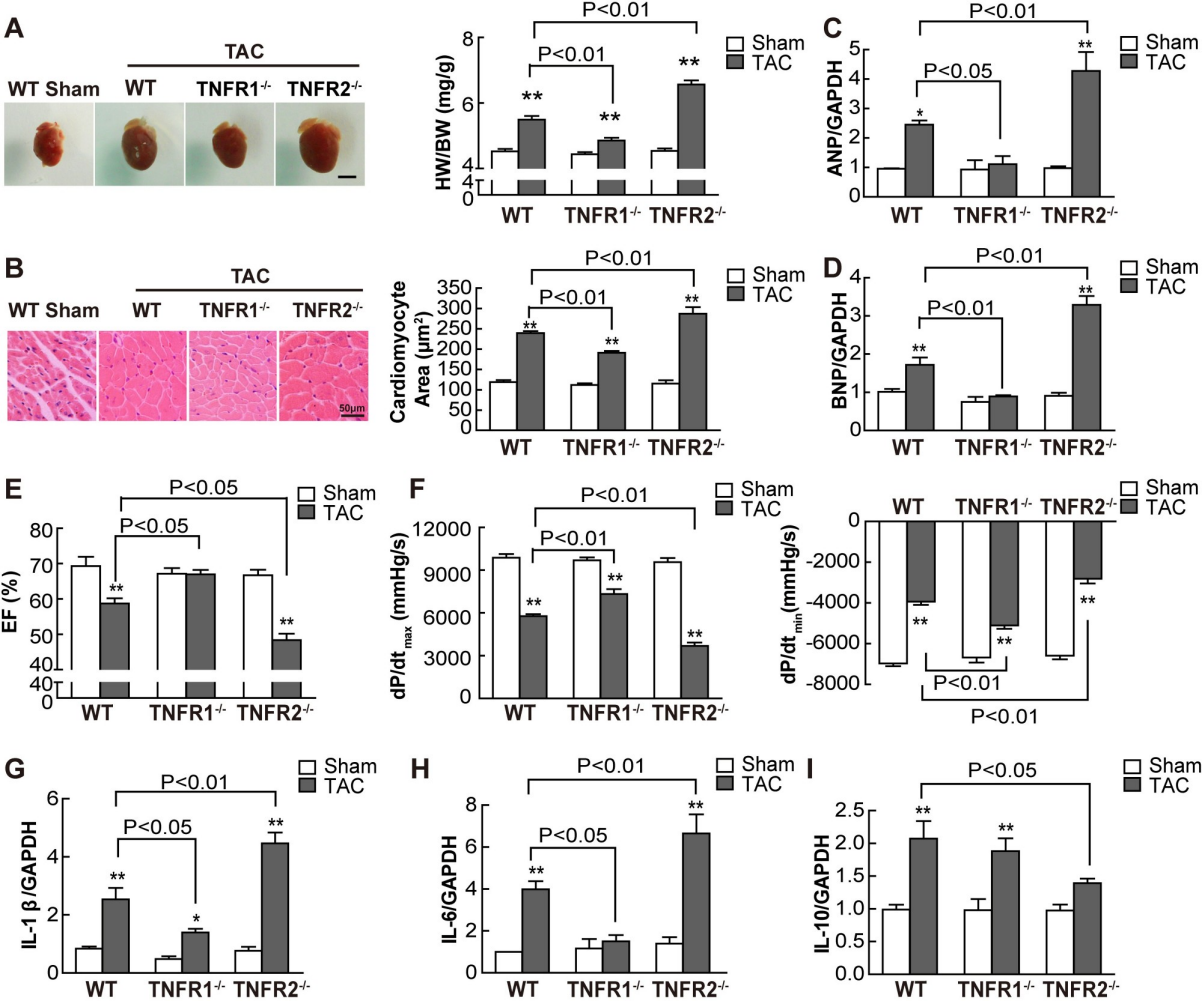
TNFR1 and TNFR2 differentially modulated cardiac hypertrophy and inflammation. WT, TNFR1^-/-^, and TNFR2^-/-^ mice were subjected to pressure overload for 2 weeks by TAC, and sham-operated mice served as controls (*n* = 6 per group). (A) Heart size and quantitative data of HW/BW ratio (mg/g). Scale bar, 3 mm. (B) Representative hematoxylin and eosin-stained myocardial sections (200×) and quantitative data of myocyte area. Quantitative RT-PCR analysis of *ANP* (C), *BNP* (D), *IL-1β* (G), *IL-6* (H), and *IL-10* (I) in myocardial tissues (*n* = 3 to 5 per group). (E) EF. (F) LV +dP/dt and LV −dP/dt were determined. *P < 0.05, **P < 0.01, ***P < 0.001 versus sham. Individual data are included in S1 Data. ANP, atrial natriuretic peptide; BNP, brain natriuretic peptide; dP/dt_max_, peak instantaneous rate of left ventricular pressure increase; dP/dt_min_, peak instantaneous rate of left ventricular pressure increase decline; EF, ejection fraction; GAPDH, glyceraldehyde 3-phosphate dehydrogenase; HW/BW, heart weight to body weight; IL, interleukin; LV, left ventricle; RT-PCR, real-time PCR; TAC, transverse aortic constriction; TNFR, TNF receptor; WT, wild-type.

To investigate the effects of TNFR1 and TNFR2 on cardiac hypertrophy, TNFR1- and TNFR2-KO mice were used for TAC-induced cardiac hypertrophy with 27G needle. TAC surgery significantly elevated velocity and echocardiography-derived trans-TAC pressure gradients to a similar extent in WT, TNFR1-, and TNFR2-KO mice (S1A–S1C Fig). The survival after 8-h perioperative period was 75% in WT, 85% in TNFR1-KO, and 61% in TNFR2-KO mice, respectively, and the animals died within 7 days (S1D Fig). The remaining survived mice were killed at 2 and 4 weeks after TAC, respectively. At 2 weeks, TNFR2 KO significantly increased TAC-induced enlargement of heart size and the ratio of heart weight to body weight (HW/BW) (Fig 1A). This promoting effect on cardiac hypertrophy was further confirmed by measurement of myocyte size (Fig 1B) and increased gene expression of *ANP* and *BNP*, 2 markers of cardiac hypertrophy (Fig 1C and 1D). In addition, TNFR2 KO markedly increased TAC-induced left ventricular hypertrophy, manifested as enhanced LV mass normalized to body weight, LVPW,d, LVAW,d, and LVID,d (S1 Table) and thus exacerbated cardiac dysfunction, including decreased EF (Fig 1E) and FS (S1 Table), increased LVEDP, and reduced LVESP (S1 Table), dP/dt_max_, and dP/dt_min_ (Fig 1F), and promoted cardiac fibrosis (S1E Fig) compared with those in WT mice. Furthermore, TNFR2 KO also facilitated TAC-induced transcription of the pro-inflammatory cytokines *IL*-*1β* and *IL-6*, but reduced *IL-10* mRNA transcription (Fig 1G–1I). In contrast, TNFR1 KO significantly ameliorated TAC-induced cardiac hypertrophy, improved cardiac function, and decreased cardiac fibrosis and pro-inflammatory cytokine transcription. These data suggested that TNFR1 aggravated, whereas TNFR2 alleviated TAC-induced cardiac hypertrophy, dysfunction, and production of pro-inflammatory cytokines.

**Fig 2:**
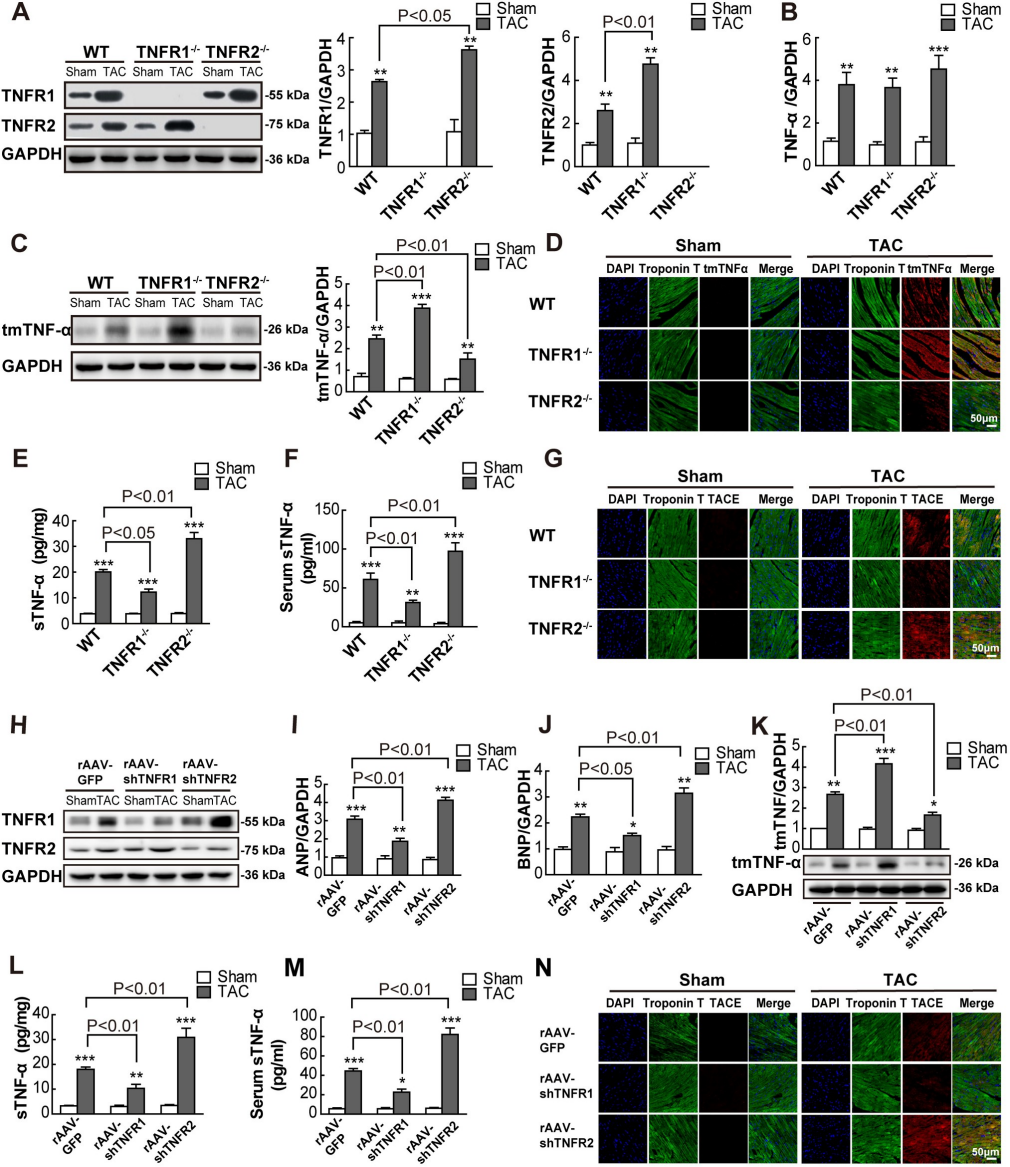
tmTNF-α processing was involved in the opposing effects of TNFR1 and TNFR2 on TAC-induced cardiac hypertrophy. WT, TNFR1^-/-^, and TNFR2^-/-^ mice were subjected to overload pressure for 2 weeks by TAC, and sham-operated mice served as controls. (A and C) Representative western blots of TNFR1, TNFR2, and tmTNF-α in myocardial tissues and quantitative data. (B) Quantitative RT-PCR analysis of *TNF-α* (*n* = 5 per group). (D and G) Representative images of indirect fluorescence costaining for troponin T and tmTNF-α or TACE on myocardial sections (400×). (E and F) sTNF-α concentrations in heart homogenates and serum detected by ELISA (*n* = 4 to 5 per group). (H–N) BALB/c mice were injected via the tail vein with rAAV-shTNFR1 or rAAV-shTNFR2 (1 × 10^11^ virion particles). rAAV-GFP served as a control. After 2 weeks, the mice were subjected to sham operation or TAC for 14 days (*n* = 6 per group). (H and K) Representative western blots for TNFR1, TNFR2, and tmTNF-α in myocardial tissues and quantitative data for tmTNF-α. (I and J) Quantitative RT-PCR analysis of *ANP* and *BNP* (*n* = 5 per group). (L and M) Concentrations of sTNF-α in heart homogenates and serum determined by ELISA (*n* = 5 per group). (N) Representative images of fluorescence immunostaining for TACE and troponin T in myocardial sections (400×). *P < 0.05, **P < 0.01, ***P < 0.001 versus sham. See individual data at S1 Data and underlying raw images at S1 Raw Images. ANP, atrial natriuretic peptide; BNP, brain natriuretic peptide; DAPI, 4′,6-diamidino-2-phenylindole; ELISA, enzyme-linked immunosorbent assay; GAPDH, glyceraldehyde 3-phosphate dehydrogenase; GFP, green fluorescent protein; RT-PCR, real-time PCR; sTNF-α, soluble TNF-α; TAC, transverse aortic constriction; TACE, TNF-α-converting enzyme; tmTNF-α, transmembrane tumor necrosis factor-alpha; TNFR, TNF receptor; WT, wild-type.

To elucidate which form of TNF-α mediated the opposite effects of the 2 TNF receptors, we used an antibody specific to tmTNF-α but not to sTNF-α [23] to detect expression levels of tmTNF-α in ventricular tissue. Additionally, ELISAs were used to detect concentrations of sTNF-α. Compared with sham controls, the expression of TNFR1, TNFR2, or both in myocardial tissue and cardiomyocytes was substantially up-regulated at 2 weeks after TAC in TNFR2-KO, TNFR1-KO, and WT mice, respectively (Fig 2A, S2A and S2B Fig). Interestingly, TAC induced *TNF-α* transcription (Fig 2B), tmTNF-α expression in myocardial tissues and cardiomyocytes, as detected by western blotting (Fig 2C) and indirect immunofluorescence (Fig 2D, S2C Fig), and sTNF-α secretion in heart homogenates and serum (Fig 2E and 2F). TNFR1 KO markedly increased TAC-induced tmTNF-α expression in myocardial tissue and cardiomyocytes, but decreased sTNF-α release, suggesting that the interaction between tmTNF-α and TNFR2 may contribute to the beneficial effects of TNFR1 KO. Conversely, TNFR2 KO reduced tmTNF-α expression but elevated sTNF-α secretion, indicating that the interaction of sTNF-α with TNFR1 mediated the detrimental effects of TNFR2 KO. However, TNFR1 KO or TNFR2 KO alone did not significantly affect TAC-induced *TNF-α* mRNA transcription (Fig 2B), indicating that both forms of TNF-α were posttranscriptionally regulated by either type of TNFR KO.

Therefore, we evaluated the expression of TACE, an enzyme responsible for tmTNF-α shedding, in myocardial cells by indirect immunofluorescence. Consistent with the results of tmTNF-α processing, TAC-induced TACE expression was markedly decreased in TNFR1-KO mice, but significantly enhanced in TNFR2-KO mice (Fig 2G, S2D Fig). To exclude the effects of systemic TNFR KO, we injected mice with rAAV9-shRNA to specifically knockdown TNFR1 or TNFR2 expression in myocardial tissue and cardiomyocytes (Fig 2H, S2E and S2F Fig). Similar opposite effects of TNFR1 and TNFR2 on TAC-induced cardiac hypertrophy and tmTNF-α processing were observed (S2 Table, Fig 2I–2N, S2G Fig). Moreover, the similar phenomena took place again at 4 weeks, later stage of TAC-induced cardiac hypertrophy, ventricular remodeling, fibrosis, and HF in WT, TNFR1-, and TNFR2-KO mice (S3A–S3L Fig, S3 Table). These data indicate that the increased expression of tmTNF-α, as a result of reduced tmTNF-α shedding, may be involved in mediating the protective effects of TNFR2 on TAC-induced cardiac hypertrophy.

### tmTNF-α may be a primary ligand for TNFR2 to mediate its protective effects on ISO-induced hypertrophy of H9C2 myocardial cells and primary cardiomyocytes

**Fig 3:**
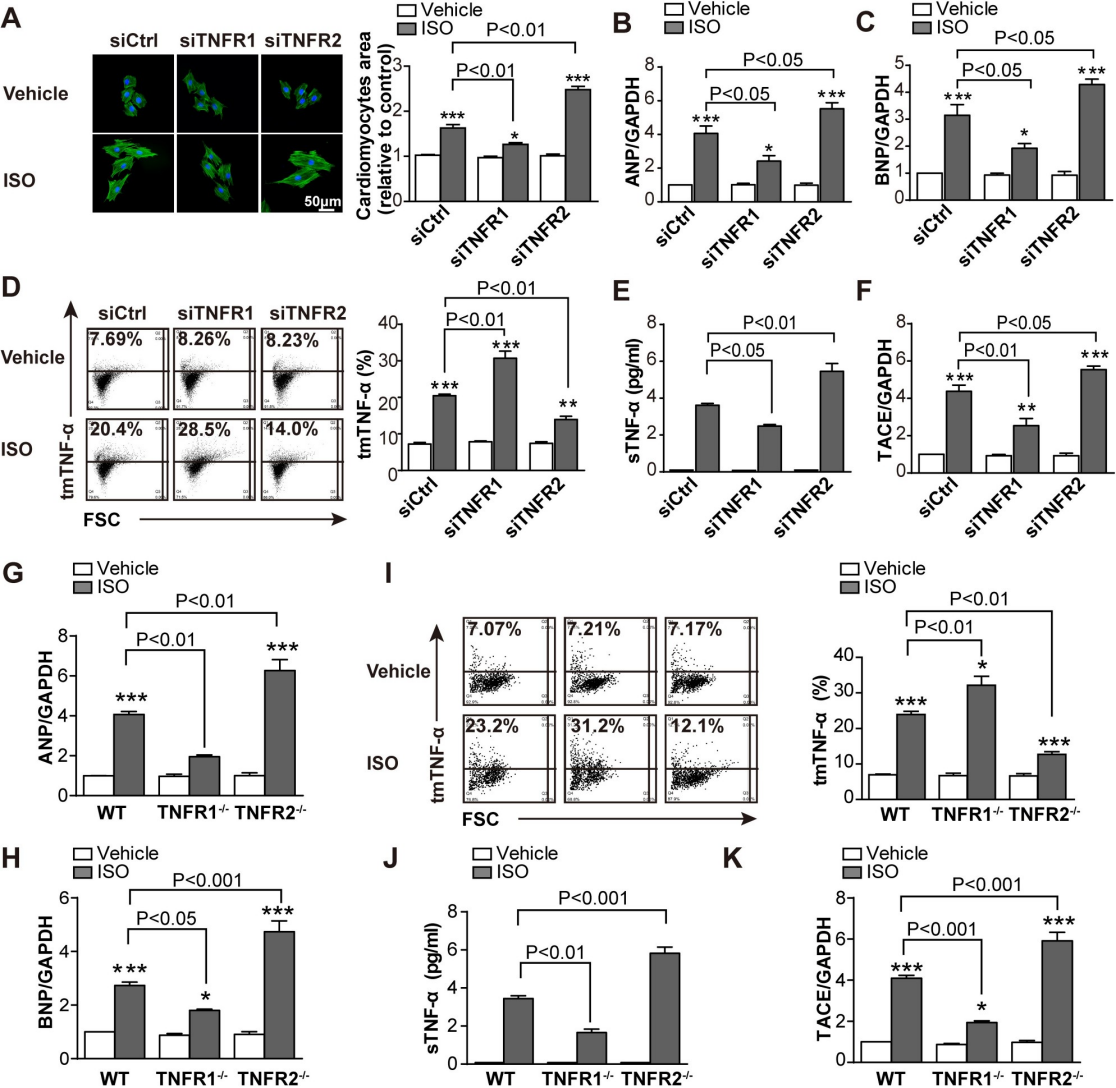
Increased tmTNF-α expression mediated the beneficial effects of TNFR1 KD/KO. ISO (10 μM) was added to H9C2 cells for 24 h after a 24-h transfection with siRNA targeting TNFR1 or TNFR2 or to primary cardiomyocytes from WT, TNFR1-KO, or TNFR2-KO mice. DMSO served as a vehicle control. (A) Representative images of H9C2 cells stained with Actin-Trakcer Green (200×) and quantitative data of the cell surface area. Scale bar, 50 μm. Quantitative RT-PCR analysis of *ANP* (B and G), *BNP* (C and H), and *TACE* (F and K). (D and I) Representative cytograms and quantitative data for tmTNF-α expression in cardiomyocytes detected by flow cytometry. (E and J) sTNF-α levels in supernatants of H9C2 or primary cardiomyocytes determined by ELISA. All quantitative data represent the means ± SEs of at least 3 independent experiments. *P < 0.05, **P < 0.01, ***P < 0.001 versus vehicle. Find individual data at S1 Data. ANP, atrial natriuretic peptide; BNP, brain natriuretic peptide; ELISA, enzyme-linked immunosorbent assay; GAPDH, glyceraldehyde 3-phosphate dehydrogenase; ISO, isoproterenol; KO, knockout; KD, knockdown; RT-PCR, real-time PCR; siRNA, small interfering RNA; sTNF-α, soluble TNF-α; TACE, TNF-α-converting enzyme; tmTNF-α, transmembrane TNF-α; TNFR, TNF receptor; WT, wild-type.

To directly observe regulation of TNF-α processing in vitro following KD of TNFR1 or TNFR2 in myocardial cells in ISO-induced hypertrophy, we used siRNA to silence the corresponding genes in H9C2 myocardial cells (S4A–S4D Fig). Consistent with the in vivo results, ISO-induced hypertrophy, including the surface area of myocardial cells (Fig 3A) and transcription of *ANP* (Fig 3B) and *BNP* (Fig 3C), was markedly enhanced by TNFR2 KD but reduced by TNFR1 KD. In addition, similar changes in tmTNF-α processing were also observed in this in vitro model, showing enhanced ISO-induced tmTNF-α expression and reduced sTNF-α secretion with down-regulation of TACE by TNFR1 KD but decreased tmTNF-α expression and elevated sTNF-α release with up-regulation of TACE by TNFR2 KD (Fig 3D–3F). We isolated primary myocardial cells from TNFR1- or TNFR2-KO mice and found the similar results in ISO-induced cardiac hypertrophy (Fig 3G–3K). Notably, ISO-induced transcription of *TNF-α* remained unchanged by KD or KO of TNFR (S4E and S4F Fig). These data strongly suggested the possibility that tmTNF-α, binding to TNFR2, may be involved in the beneficial effects of TNFR1 KD/KO, whereas sTNF-α, binding to TNFR1, may be associated with the detrimental effects of TNFR2 KD/KO.

### Suppression of TACE to increase endogenous tmTNF-α expression alleviated TAC-induced cardiac hypertrophy

**Fig 4:**
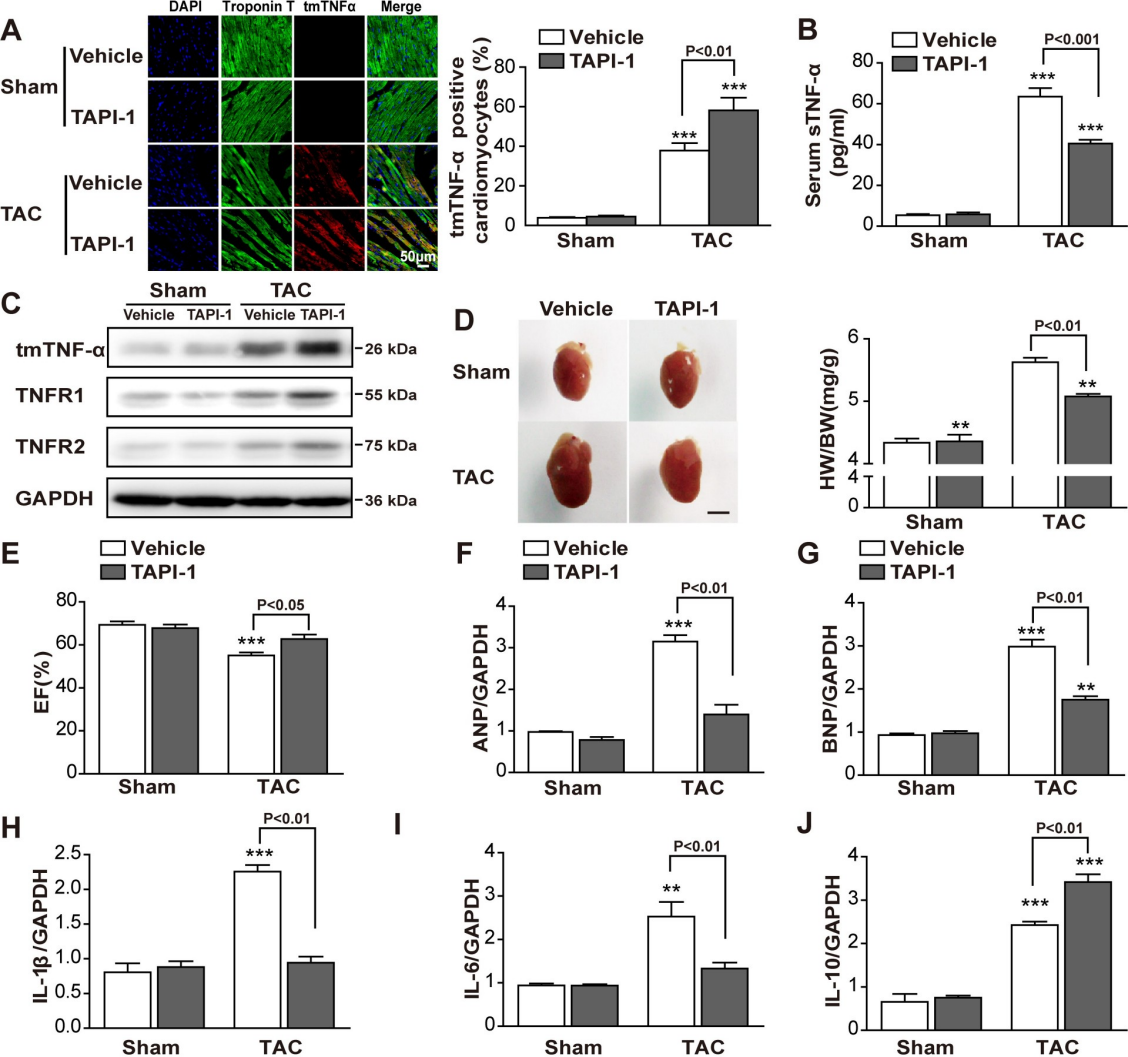
Suppression of TACE alleviated TAC-induced cardiac hypertrophy. TAPI-1 (8 mg/mL) was administered to WT mice by implantation of osmotic pumps (0.25 μL/h) directly after TAC or sham operation (*n* = 6 per group). (A) Representative images of tmTNF-α fluorescence immunostaining on myocardial sections (400×) and quantitative data (*n* = 5 per group). (B) Serum levels of sTNF-α detected by ELISA (*n* = 3 to 4 per group). (C) Western blot analysis of tmTNF-α, TNFR1, and TNFR2. (D) Heart size and quantitative data of HW/BW ratio (mg/g). Scale bar, 3 mm. (E) Assessment of EF. (F–J), Quantitative RT-PCR analysis of *ANP*, *BNP*, *IL-1β*, *IL-6*, and *IL-10* in myocardial tissues (*n* = 3 to 4 per group). **P < 0.01, ***P < 0.001 versus corresponding group in sham. Individual data can be found in S1 Data and underlying raw images in S1 Raw Images. ANP, atrial natriuretic peptide; BNP, brain natriuretic peptide; DAPI, 4′,6-diamidino-2-phenylindole; EF, ejection fraction; ELISA, enzyme-linked immunosorbent assay; GAPDH, glyceraldehyde 3-phosphate dehydrogenase; HW/BW, heart weight to body weight; IL, interleukin; RT-PCR, real-time PCR; sTNF-α, soluble TNF-α; TAC, transverse aortic constriction; TACE, TNF-α-converting enzyme; TAPI-1, TNF-α protease inhibitor-1; tmTNF-α, transmembrane TNF-α; TNFR, TNF receptor; WT, wild-type.

Our results suggested that the beneficial of TNFR1KD/KO was associated with increased tmTNF-α expression, as a result of down-regulating TACE expression and thus suppressing tmTNF-α processing. We used TACE inhibitor TAPI-1 to block tmTNF-α processing and increase tmTNF-α expression to observe whether TAC-induced cardiac hypertrophy could be relieved in WT mice. Consistently, inhibition of TACE by TAPI-1 significantly increased TAC-induced tmTNF-α expression in cardiomyocytes and myocardial tissue (Fig 4A and 4C) and markedly reduced serum levels of sTNF-α (Fig 4B). Because TACE is also responsible for ectodomain shedding of TNFR1 and TNFR2, we detected the effects of TAPI-1 on TNFR expression. Indeed, TAPI-1 significantly enhanced TAC-induced expression of both TNFR1 and TNFR2 (Fig 4C). Importantly, up-regulation of tmTNF-α by TAPI-1 significantly alleviated TAC-induced cardiac hypertrophy and cardiac dysfunction, manifested by reduced heart size and HW/BW ratio (Fig 4D), increased EF (Fig 4E), and decreased TAC-induced transcription of *ANP* and *BNP* (Fig 4F and 4G). Moreover, the increased expression of tmTNF-α by TAPI-1 inhibited *IL-1β* and *IL-6* expression (Fig 4H and 4I) and enhanced *IL-10* expression (Fig 4J). Inhibition of TACE-mediated tmTNF-α processing by TAPI-1 ameliorated TAC-induced cardiac hypertrophy and the production of inflammatory cytokines, indicating that increased tmTNF-α expression and decreased sTNF-α release contributed to the beneficial effect of suppressing tmTNF-α processing in pressure overload-induced cardiac hypertrophy.

### Exogenous tmTNF-α directly protected myocardiocytes from ISO-induced hypertrophy and promoted TACE expression via TNFR2

**Fig 5:**
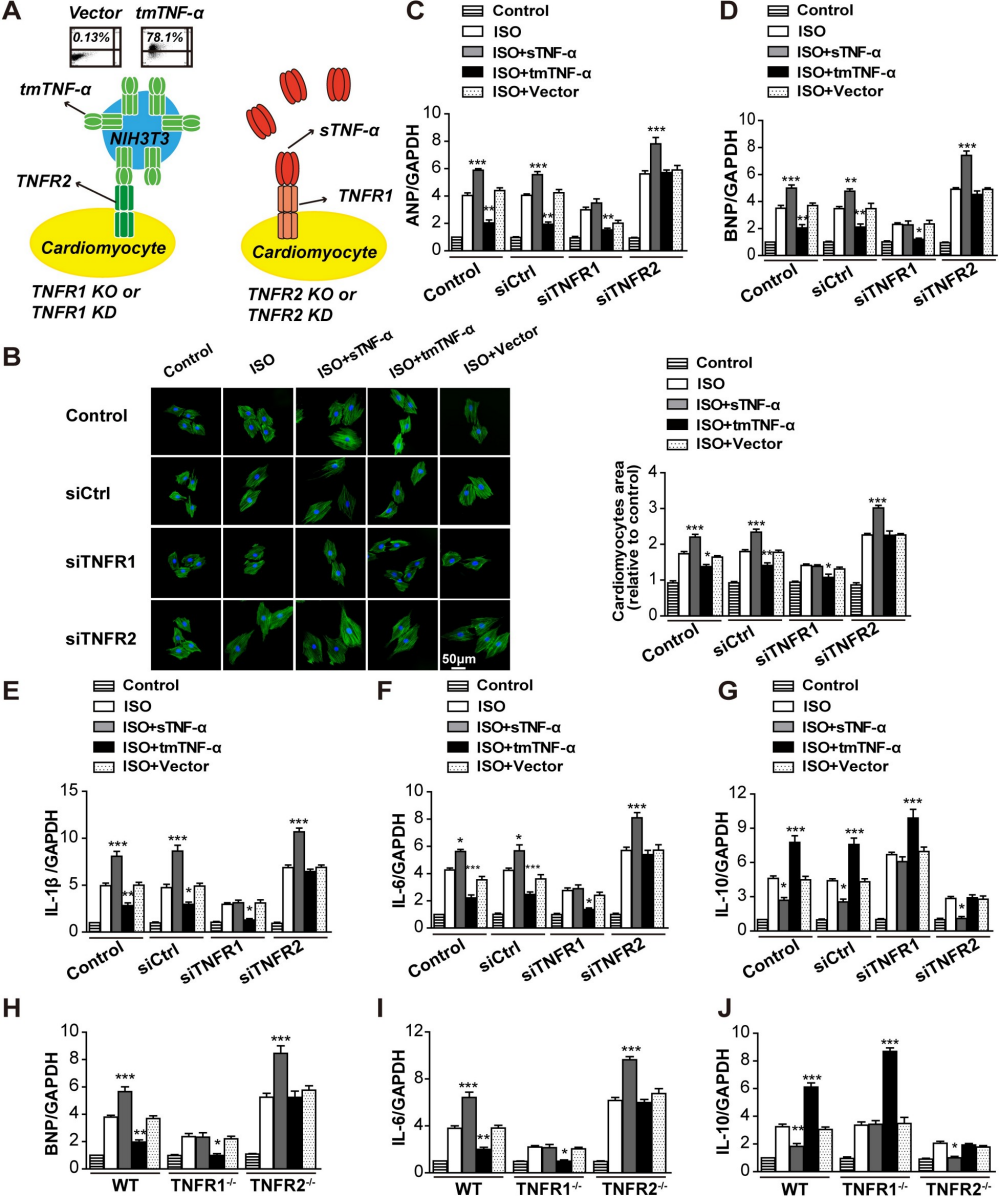
Exogenous tmTNF-α directly protected myocardiocytes from ISO-induced hypertrophy via TNFR2. (A) The schema of experimental design: Exogenous sTNF-α (20 ng/mL) or tmTNF-α on fixed NIH3T3 cells was added to H9C2 cells transfected with siRNA targeting TNFR1 or TNFR2 or to primary cardiomyocytes from WT, TNFR1-KO, or TNFR2-KO mice at an effector/target ratio of 10:1 and incubated for 24 h in the presence of ISO (10 μM). Vector-transfected NIH3T3 cells served as a control. Ectopic expression of tmTNF-α on the surface of NIH3T3 cells detected by flow cytometry. (B) Representative images of H9C2 cells stained with Actin-Tracker Green (200×) and quantitative data of the cell surface area, as analyzed using Image-Pro Plus 6.0 software. Scale bar, 50 μm. Quantitative RT-PCR analysis of *ANP* (C), *BNP* (D and H), *IL-1β* (E), *IL-6* (F and I), and *IL-10* (G and J). All quantitative data represent means ± SEs of 5 independent experiments. *P < 0.05, **P < 0.01, ***P < 0.001 versus corresponding ISO. Individual data are included in S1 Data. ANP, atrial natriuretic peptide; BNP, brain natriuretic peptide; GAPDH, glyceraldehyde 3-phosphate dehydrogenase; IL, interleukin; ISO, isoproterenol; KO, knockout; KD, knockdown; RT-PCR, real-time PCR; siRNA, small interfering RNA; sTNF-α, soluble TNF-α; tmTNF-α, transmembrane TNF-α; TNFR, TNF receptor; WT, wild-type.

Our results in vivo and in vitro showing the beneficial effects of TNFR1 KD/KO with increased tmTNF-α expression indicated that tmTNF-α might mediate the protective effects of TNFR2. To test this hypothesis, we directly treated H9C2 cells or primary cardiomyocytes, as target cells, with exogenous tmTNF-α expressed on fixed NIH3T3 cells at an E/T ratio of 10:1 and sTNF-α (20 ng/mL; Fig 5A). In contrast to the aggravating effects of sTNF-α, tmTNF-α displayed protective effects, resulting in reduced ISO-induced hypertrophy of myocardial cells (Fig 5B); decreased *ANP* (Fig 5C), *BNP* (Fig 5D and 5H), *IL-1β* (Fig 5E), and *IL-6* (Fig 5F and 5I) transcription, and increased *IL-10* transcription (Fig 5G and 5J). Interestingly, the beneficial effects of tmTNF-α were totally abolished by KD or KO of TNFR2 rather than TNFR1, indicating that tmTNF-α exerted protective effects via TNFR2. In contrast, the promoting effects of sTNF-α on ISO-induced hypertrophy of myocardial cells and the induction of pro-inflammatory cytokines were entirely obstructed by TNFR1 KD/KO, implying that sTNF-α displayed pathogenic effects via TNFR1. For the anti-inflammatory cytokine IL-10, the promoting effects of tmTNF-α were prevented by TNFR2 KD/KO, but the inhibitory effects of sTNF-α were abolished by TNFR1 KD/KO (Fig 5G and 5J).

Apoptosis is a very important mechanism for decompensation of pressure overload–induced cardiac hypertrophy and HF [24]. Our results showed that TAC induced a few of apoptotic cells detected by TUNEL at 2 weeks, which could be significantly enhanced at 4 weeks in WT mice (S5A and S5B Fig). Importantly, TAC-induced apoptosis was remarkably inhibited by TNFR1 KO, but facilitated by TNFR2 KO, suggesting that TNFR1 signaling mediated apoptosis. sTNF-α causes apoptosis of myocardial cells [25,26], but the role of tmTNF-α is unknown. We found that tmTNF-α-overexpressed in fixed 3T3 cells stimulated proliferation, instead of apoptosis, in H9C2 cells, which could be effectively prevented by TNFR2 KD (S5E and S5F Fig). In contrast, sTNF-α induced about 58% apoptosis and was highly cytotoxic to H9C2 cells (about 73%), which could be blocked by TNFR1 KD (S5C and S5D Fig). These data suggest that in contrast to sTNF-α-induced apoptosis through TNFR1, tmTNF-α promotes proliferation of cardiomyocytes via TNFR2, indicating a mechanism involved in the cardioprotective effect of tmTNF-α.

**Fig 6:**
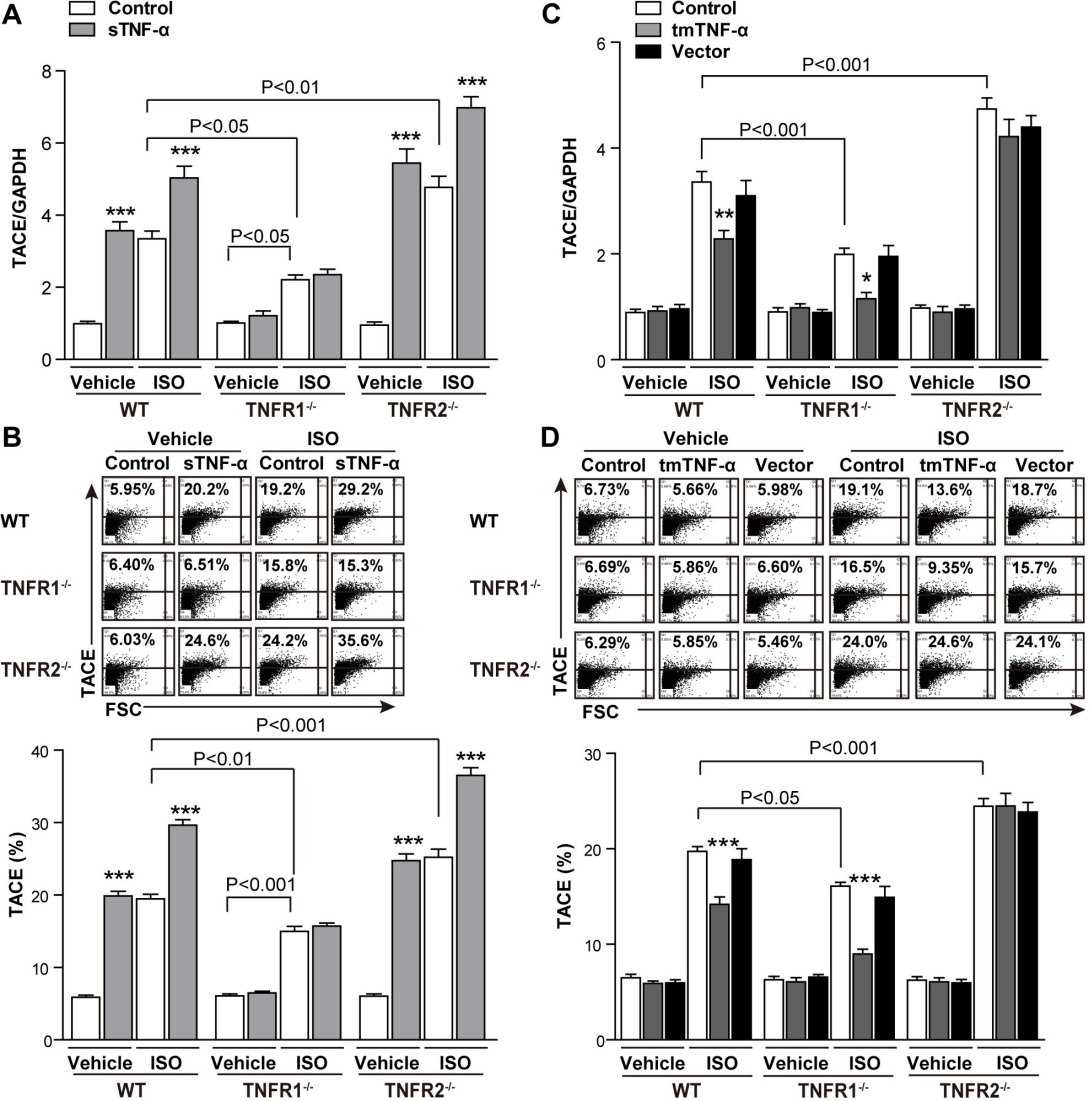
Down-regulation of TACE by tmTNF-α via TNFR2. Primary cardiomyocytes from WT, TNFR1-KO, or TNFR2-KO mice were treated with ISO (10 μM) for 24 h in the presence or absence of exogenous sTNF-α (20 ng/mL) and tmTNF-α on fixed NIH3T3 cells (at an effector/target ratio of 10:1). Vector-transfected NIH3T3 cells served as a control. (A and C) Quantitative RT-PCR analysis of *TACE*. (B and D) Representative cytograms and quantitative data for TACE expression on the cell surface of cardiomyocytes detected by flow cytometry. All quantitative data represent means ± SEs of 5 independent experiments. *P < 0.05, **P < 0.01, ***P < 0.001 versus corresponding control. See individual data at S1 Data. FSC, Forward scatter; GAPDH, glyceraldehyde 3-phosphate dehydrogenase; ISO, isoproterenol; KO, knockout; KD, knockdown; RT-PCR, real-time PCR; sTNF-α, soluble TNF-α; TACE, TNF-α-converting enzyme; tmTNF-α, transmembrane TNF-α; TNFR, TNF receptor; WT, wild-type.

As pressure overload induces mechanical stress in myocardial cells, a 48-h mechanical stretch was used. We found that exposure of H9C2 cells to mechanical stretch not only induced mRNA transcription of *ANP* and *BNP* (S6A and S6B Fig) but also induced *TACE* transcription and the protein expression (S6C and S6D Fig). In line with TAC- or ISO-induced TACE expression, TNFR1 KD decreased, but TNFR2 KD increased stretch-induced *TACE* mRNA transcription (S6E Fig). Because of regulating TACE expression by TNFR KD/KO, both forms of TNF-α, as TNFR ligands, must be involved in this event. sTNF-α has been reported to induce TACE expression, promoting soluble TNFR release [27]. Based on down-regulation of TACE in TNFR1-KO mice with increased tmTNF-α, we hypothesized that tmTNF-α may suppress TACE expression via TNFR2. As expected, sTNF-α alone induced TACE transcription and protein expression and enhanced ISO-induced TACE expression in cardiomyocytes from WT mice; both of them were completely blocked by TNFR1 KO (Fig 6A and 6B). In contrast, tmTNF-α significantly inhibited ISO-induced TACE transcription and protein expression in WT and TNFR1-KO cardiomyocytes, although tmTNF-α alone had no effect. This effect of tmTNF-α was completely abolished by TNFR2 KO (Fig 6C and 6D). The inhibitory effect of tmTNF-α on TACE expression represented another mechanism underlying the protective effects of TNFR2.

### tmTNF-α displays the protective effects via TNFR2 through activating AKT pathway and suppressing NF-κB pathway

**Fig 7:**
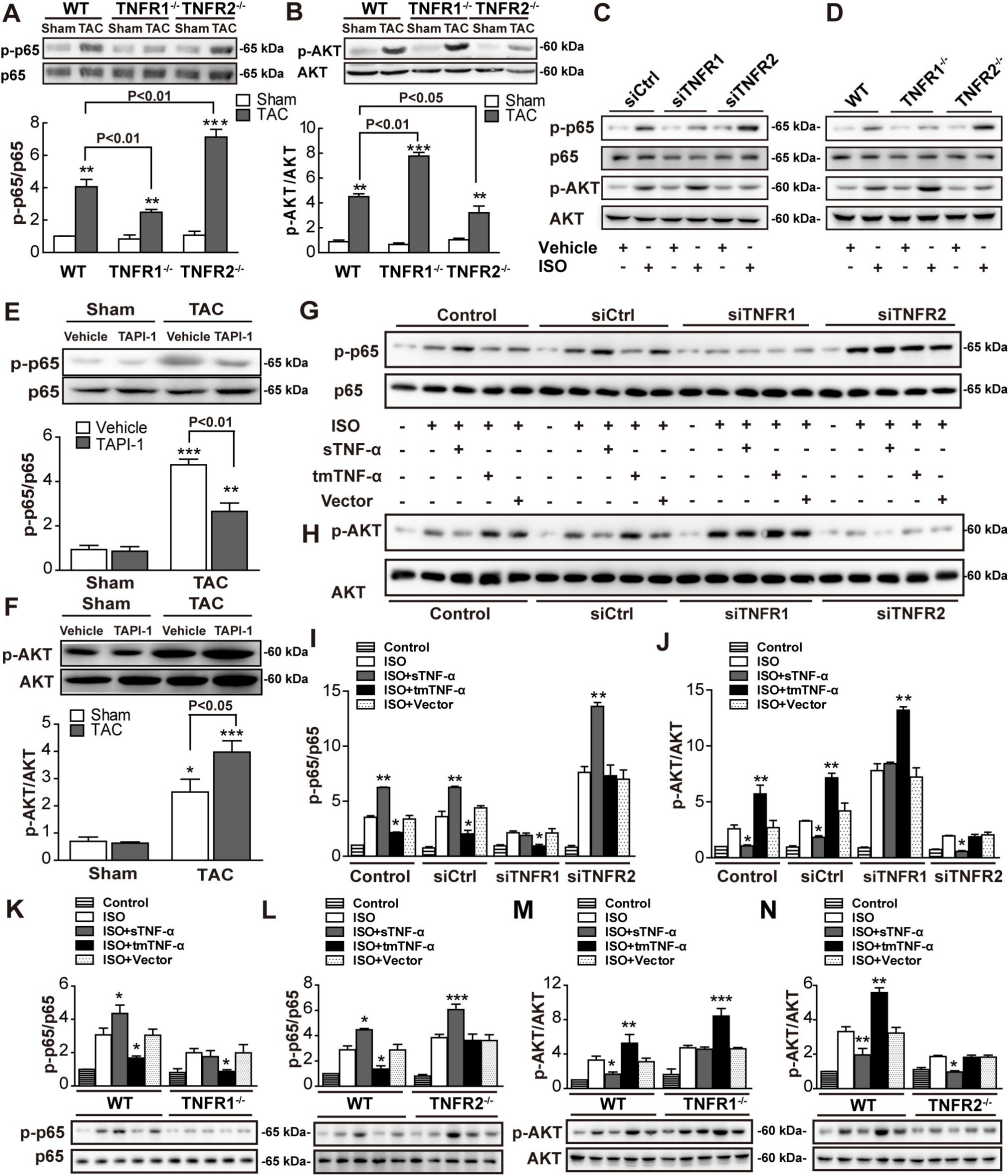
tmTNF-α promoted pressure overload–induced AKT activation but inhibited the NF-κB pathway via TNFR2. Representative immunoblots and quantitative data for phosphorylation levels of NF-κB p65 and AKT in LV tissues from WT, TNFR1-KO, and TNFR2-KO mice 2 weeks after TAC operation (A and B) and treatment with TAPI-1 (8 mg/mL) (E and F). (C and D) Representative immunoblots for phosphorylation levels of NF-κB p65 and AKT in H9C2 cells transfected with siRNA for TNFR or in primary cardiomyocytes from TNFR-KO mice, treated with ISO (10 μM) for 24 h. Representative immunoblots and quantitative data for phosphorylation levels of NF-κB p65 and AKT in H9C2 cells transfected with siRNA for TNFR (G–J) or in primary cardiomyocytes from TNFR-KO mice (K–N), treated with ISO (10 μM) and sTNF-α (20 ng/mL) or tmTNF-α on fixed NIH3T3 cells at an effector/target ratio of 10:1. All quantitative data represent means ± SEs of at least 3 independent experiments. *P < 0.05, **P < 0.01, ***P < 0.001 versus shame (A and B) or vehicle (E and F) or ISO (I–N). See individual data at S1 Data and underlying raw images at S1 Raw Images. ISO, isoproterenol; KO, knockout; LV, left ventricle; NF-κB, nuclear factor kappa B; siRNA, small interfering RNA; sTNF-α, soluble TNF-α; TAC, transverse aortic constriction; tmTNF-α, transmembrane TNF-α; TNFR, TNF receptor; WT, wild-type.

To explore the signaling pathways mediated by tmTNF-α in pressure overload–induced cardiac hypertrophy, we examined activation of the NF-κB and AKT pathways in myocardial tissues from TNFR-KO mice in vivo or in cardiomyocytes in vitro by western blotting. TAC induced activation of NF-κB and AKT pathways. TNFR2 KO significantly augmented, but TNFR1 KO markedly suppressed TAC-induced phosphorylation of NF-κB p65 (Fig 7A) in myocardial tissue compared with that in WT mice. In contrast, TNFR2 KO inhibited, but TNFR1 KO enhanced TAC-induced phosphorylation of AKT (Fig 7B). Similar results were observed in ISO-induced cardiac hypertrophy in vitro using H9C2 cells or primary cardiomyocytes by KD or KO of TNFR1 or TNFR2 (Fig 7C and 7D). Importantly, using TACE inhibitor TAPI-1 to increase tmTNF-α expression significantly suppressed TAC-induced NF-κB activation (Fig 7E) but increased AKT phosphorylation (Fig 7F) in myocardial tissues from WT mice. These data indicated that the protective effects of tmTNF-α may be mediated by enhancing AKT activation and suppressing the NF-κB pathway via TNFR2.

To test this hypothesis, we added exogenous sTNF-α or tmTNF-α on NIH3T3 cells to H9C2 cells or to primary cardiomyocytes at a ratio of 10:1 and found that tmTNF-α inhibited, but sTNF-α promoted ISO-induced activation of the NF-κB pathway (Fig 7G, 7I, 7K, and 7L); in contrast, tmTNF-α enhanced, but sTNF-α suppressed ISO-induced activation of the AKT pathway (Fig 7H, 7J, 7M, and 7N). The promoting effect of tmTNF-α on the AKT pathway and the suppressive effect of tmTNF-α on the NF-κB pathway could be completely abolished by TNFR2 KD/KO. Conversely, the inhibitory effect of sTNF-α on AKT and the stimulatory effect of sTNF-α on NF-κB could be entirely blocked by TNFR1 KD/KO. These data implied that tmTNF-α displayed protective effects through activation of AKT and inhibition of NF-κB mainly via TNFR2, whereas sTNF-α exerted pathogenic effects through inhibition of AKT and activation of NF-κB via TNFR1.

## Discussion

In the present study, we demonstrated, for the first time, that tmTNF-α was a major ligand for TNFR2-mediated cardioprotective effects because tmTNF-α expression was increased by TNFR1 KD/KO, and addition of exogenous tmTNF-α attenuated pressure overload–induced cardiac hypertrophy and inflammation through activation of AKT and inhibition of NF-κB via TNFR2. Using a TACE inhibitor to increase tmTNF-α expression attenuated TAC-induced cardiac hypertrophy and inflammation, highlighting this molecule as a promising target for the treatment of pressure overload–induced cardiac hypertrophy.

Consistent with previous studies [18,28], we showed that pressure overload–induced cardiac hypertrophy and inflammation were worsened in TNFR2-KO mice, but improved in TNFR1-KO mice, indicating harmful effects via TNFR1 and beneficial effects via TNFR2. However, 1 report, which used a different mouse strain and experimental system, showed that TNFR2 KO did not affect this pathological process [29]. Moreover, the specific ligands binding to the different TNFRs, which displayed opposite functions, have not been clarified. In the present study, we primarily found increased tmTNF-α expression and reduced sTNF-α secretion in heart tissues following systemic TNFR1 KO or cardiac TNFR1 KD and converse changes by TNFR2 KO/KD in TAC-induced cardiac hypertrophy. This phenomenon was confirmed again in ISO-induced cardiac hypertrophy in TNFR-KD H9C2 cells and primary cardiomyocytes from TNFR-KO mice. Second, our data suggest that interaction of tmTNF-α with TNFR2 may be responsible for the anti-hypertrophic and anti-inflammatory effects of TNFR1 KO/KD, whereas the interaction of sTNF-α with TNFR1 was related to the pro-hypertrophic, pro-apoptotic and pro-inflammatory effects of TNFR2 KO/KD. This hypothesis was supported by the following evidences: (1) Using TACE inhibitor TAPI-1 to increase tmTNF-α expression in WT mice significantly improved TAC-induced cardiac hypertrophy, decreased transcription of pro-inflammatory cytokines, and enhanced *IL-10* mRNA expression. Consistent with our study, deficiency in the TACE inhibitor, tissue inhibitor of metalloproteinases-3, alone is sufficient to result in LV dilatation, cardiomyocyte hypertrophy, and contractile dysfunction with increased cardiac sTNF-α and sTNFR2 [30]. (2) Importantly, direct addition of exogenous tmTNF-α into myocardial cell culture significantly alleviated ISO-induced cardiac hypertrophy and inflammatory cytokine production, and induced proliferation, rather than apoptosis in H9C2 cells, and the beneficial effects were entirely blocked by TNFR2 KO or/and KD; in contrast, addition of sTNF-α markedly exacerbated the pathological changes and induced apoptosis, and these effects were completely abolished by TNFR1 KO or/and KD. It is well known that TNFR1 mediates most pathogenic activities of sTNF-α in cardiac hypertrophy and HF [28,29]. Our data strongly suggest that tmTNF-α mediates cardiac protection via TNFR2.

Interestingly, TAC or ISO-induced *TNF-α* transcription was not affected by TNFR1 or TNFR2 KO/KD alone in vivo or in vitro, suggesting that TNF-α was regulated posttranscriptionally. Our results originally revealed that TNFR1 KO/KD reduced TACE expression; as a result, sTNF-α release was decreased with high tmTNF expression, while TNFR2 KO/KD increased TACE expression; as a consequence, sTNF-α release was enhanced with lower tmTNF expression in heart tissues and cardiomyocytes, suggesting that TACE, an enzyme responsible for ectodomain shedding of tmTNF-α, played an important role. Since pressure overload induces mechanical stress in myocardial cells, we used a 48-h mechanic stretch to induce hypertrophy of H9C2 cells and found that mechanic stress induced TACE expression, which was inhibited by TNFR1 KD but promoted by TNFR2 KD. Importantly, we found that treatment with exogenous tmTNF-α significantly suppressed ISO-induced *TACE* transcription and protein expression; this effect could be entirely dampened by TNFR2 KD. sTNF-α has been reported to induce TACE expression to promote soluble TNFR release for buffer of itself [27], we uncovered that sTNF-α-induced TACE production or sTNF-α-promoted ISO-induced TACE expression could be completely blocked by TNFR1 KD. These data suggest that sTNF-α positively regulates, but tmTNF-α negatively regulates TACE expression via TNFR1 and TNFR2, respectively. Notably, elevated TACE expression is associated with enhanced sTNF-α levels in clinical advanced congestive HF [31] and human dilated cardiomyopathy [14]. However, tmTNF-α alone had no effect on TACE production. The reason why did TNFR KO/KD affect TACE expression after TAC or ISO treatment may be as follows: Pressure overload–induced mechanical stress up-regulated TACE expression. TNFR1 KO/KD blocked sTNF-α-mediated positive regulation leading to reduced TACE expression followed by enhanced expression of tmTNF-α that further down-regulated pressure overload–induced TACE expression via TNFR2. Conversely, TNFR2 KO/KD increased TACE expression by blockage of tmTNF-α-mediated negative regulation and promotion of sTNF-α-mediated positive regulation via TNFR1.

Furthermore, our data showed that tmTNF-α exerted beneficial effects via TNFR2 through activation of the AKT pathway, a cardioprotective signaling pathway [32–35], and inhibition of the NF-κB pathway, which is involved in cardiac hypertrophy, adverse remodeling, and inflammation [36–38]. First, we found that TAC- or ISO-induced NF-κB p65 phosphorylation was suppressed, but AKT phosphorylation was enhanced by TNFR1 KO/KD, whereas the opposite effects were observed by TNFR2 KO/KD. Second, using a TACE inhibitor to increase tmTNF-α expression resulted in suppression of TAC-induced NF-κB p65 phosphorylation and promotion of AKT phosphorylation. Third, the addition of exogenous tmTNF-α significantly suppressed ISO-induced NF-κB activation but enhanced ISO-induced AKT activation; these effects were completely blocked by TNFR2 KD/KO. Consistently, our previous study demonstrated that tmTNF-α down-regulated IL-6 and monocyte chemotactic protein-1 production via inactivation of the NF-κB pathway and promoted insulin-induced phosphorylation of insulin receptor substrate-1 and AKT pathway to sensitize adipocytes to insulin [13]. Because the AKT pathway facilitates the survival and proliferation of cardiomyocytes [39], this pathway represented a cardioprotective mechanism involving tmTNF-α. Although noncleavable tmTNF-α transgenic mice developed concentric hypertrophy [16], in our study, TNF-α expression was induced by pressure overload, rather than constitutive expression, and tmTNF-α exhibited regulatory functions.

**Fig 8:**
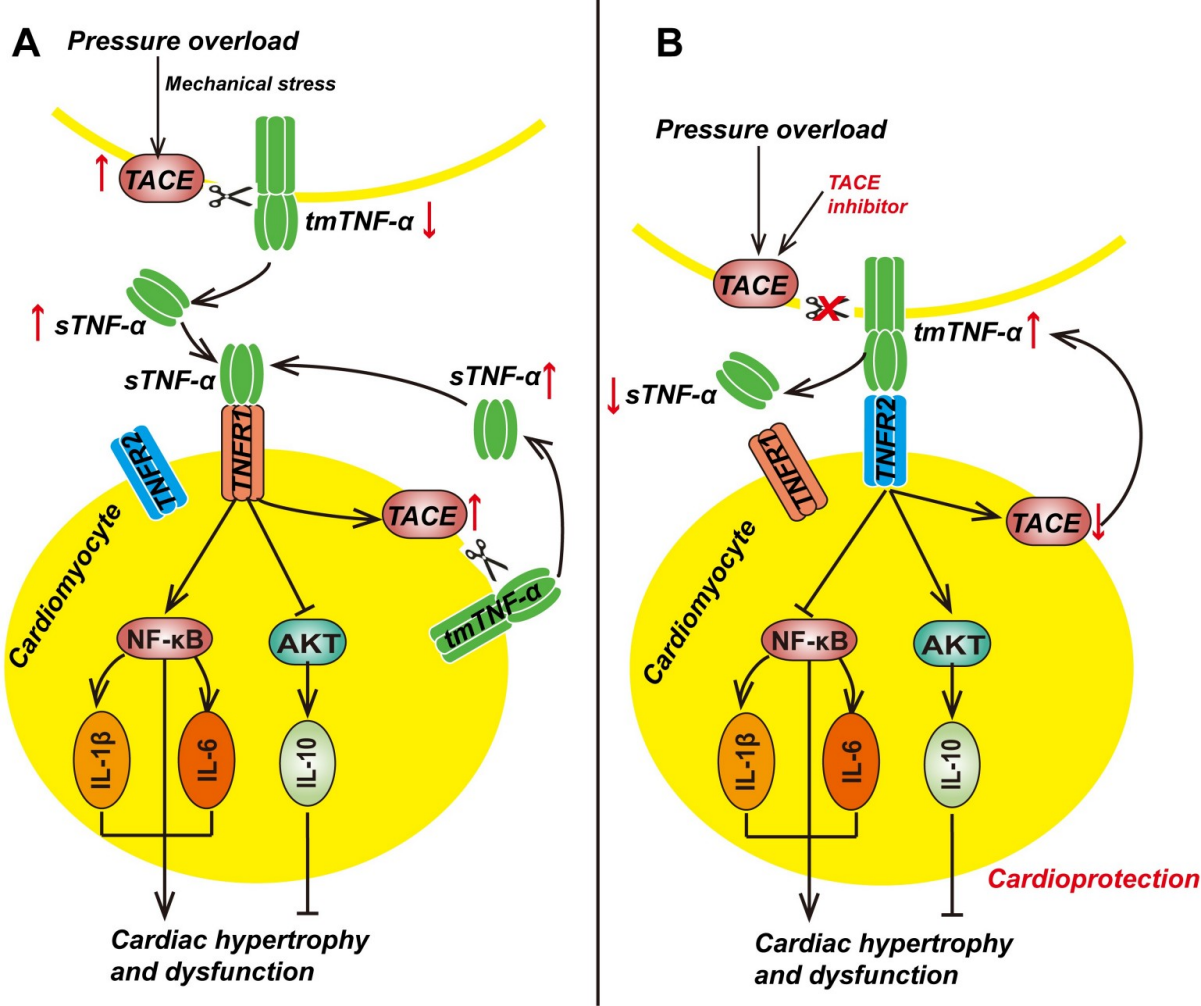
Schematic summary of the cardioprotective effect of tmTNF-α via TNFR2 on cardiac hypertrophy and inflammation. In contrast to sTNF-α that exerts prohypertrophy, and pro-inflammation through activating NF-κB pathway and inhibiting AKT pathway via TNFR1 (A), tmTNF-α displays antihypertrophy and anti-inflammation through suppressing NF-κB pathway and activating AKT pathway via TNFR2 in pressure overload–induced cardiac hypertrophy (B). In addition, mechanical stress induces TACE expression followed by enhanced release of sTNF-α that increases TACE expression via TNFR1, which cleaves tmTNF-α to produce more sTNF-α to display detrimental effects (A), whereas inhibition of TACE increases expression of tmTNF-α that down-regulates pressure overload–induced TACE expression via TNFR2, which in turn reduces tmTNF-α processing, and consequence increases tmTNF-α expression to display cardioprotective activities (B). IL, interleukin; NF-κB, nuclear factor-kappa B; sTNF-α, soluble TNF-α; TACE, TNF-α-converting enzyme; tmTNF-α, transmembrane TNF-α; TNFR, TNF receptor.

In summary, our results suggest that tmTNF-α exhibits cardioprotective effects through suppression of NF-κB and activation of the AKT pathway via TNFR2, in contrast to detrimental effects of sTNF-α via TNFR1 in pressure overload–induced cardiac hypertrophy (Fig 8). The opposing receptor-specific responses were ligand dependent [40]; hence, selective targeting of tmTNF-α processing, rather than anti-TNF therapy, may be more useful for the treatment of hypertrophy and HF by diminishing the deleterious effects of sTNF-α while enhancing the cardioprotective and anti-inflammatory effects of tmTNF-α.

## Materials and methods

### Ethics statement

Our animal studies were approved by the Institutional Animal Care and Use Committee of Tongji Medical College, Huazhong University of Science and Technology (IACUC approval number 2365).

### Animals

Male mice (10 to 14 weeks old) were used. TNFR1-KO and TNFR2-KO BALB/c mice were a kind gift from Prof. Zhihai Qin (National Laboratory of Biomacromolecules, Institute of Biophysics Chinese Academy of Sciences, Beijing, China) [41]. WT BALB/c mice were purchased from the Experimental Animal Center of Tongji Medical College. Mice were bred and maintained under specific pathogen-free conditions and confirmed by specific primer genotyping.

### TAC in mice

TAC was performed as described previously [42]]. Briefly, after anesthesia of mice with intraperitoneal administration of 50-mg/kg pentobarbital, the transverse thoracic aorta was narrowed to 0.41 mm in diameter by ligation using a 7–0 silk suture with an overlaid 27-gauge needle, followed by subsequent removal of the needle. Sham animals underwent a similar surgical procedure, except constriction of the aorta was not performed. Mice were killed at 2 and 4 weeks after the operation.

### Osmotic pump implantation and drug treatment in vivo

Alzet pumps (type 2004, 100 μl, 0.25 μl/hr; Alzet Osmotic Pumps, Cupertino, California, United States of America) were filled with a broad matrix metalloprotease (MMP)/adisintegrin and metalloproteinase (ADAM) inhibitor TAPI-1 (Enzo Life Sciences, Farmingdale, New York, USA) at a concentration of 8 mg/ml in PBS. The osmotic pumps then were kept in 0.9% saline at 37°C before implantation. After TAC surgery, osmotic pump was placed subcutaneously on the back of mice, and mice were housed individually, and the infusion lasted for 14 days.

### Echocardiography

Transthoracic echocardiography was performed utilizing a Vevo770 ultrasound machine with a 30-MHz high-frequency scan head (VisualSonics, Toronto, Canada). After inhalation anesthesia of isoflurance, a parasternal long-axis view was obtained for M-mode imaging. All measurements were made by a trained observer blinded to the experimental groups and were averaged over 5 consecutive cardiac cycles. Heart rate (HR), velocity, left ventricle (LV) mass, LV internal diameter at end-diastole (LVID,d), LV posterior wall thickness at end-diastole (LVPW,d), LV anterior wall thickness at end-diastole (LVAW,d), ejection fraction (EF), and fractional shortening (FS) of the LV were measured.

### Hemodynamics

Mice were anesthetized by intraperitoneal administration of pentobarbital at 50-mg/kg body weight, and hemodynamic variables were evaluated with a 1.4-Fr high-fidelity SPR-839 Millar pressure catheter (Millar Instruments, Houston, Texas, USA) advanced from the right carotid artery via the aortic arch into the LV at the end of the study. These hemodynamic variables included LV end-systolic pressure (LVESP), LV end-diastolic pressure (LVEDP), peak instantaneous rate of left ventricular pressure increase (dP/dt_max_), and peak instantaneous rate of left ventricular pressure increase decline (dP/dt_min_).

### Histology, collagen staining, and indirect immunofluorescence

Diseased hearts were excised, dissected free of adherent tissue, blotted dry, and weighed. A ring of LV tissue, cut at the level of the papillary muscles, was fixed in 10% buffered formalin and embedded in paraffin. The samples were cut into 4-μm sections and stained with hematoxylin and eosin for cardiomyocyte area analysis and with Sirius red for the detection of collagen. Images were viewed and captured by an Olympus BH-2 light microscope (Olympus, Tokyo, Japan) attached to a computerized imaging system. The positive cells were counted at ×200 magnification, in 5 random fields, with the Image-Pro Plus Version 6.0 software (Media Cybernetics, Bethesda, Maryland, USA).

Indirect immunofluorescence analysis was performed as described previously [42]. Briefly, heart tissue sections were incubated with primary antibodies including anti-tmTNF-α (made in house) [23], anti-TACE, anti-TNFR1, anti-TNFR2, and anti-troponin T antibodies at 37°C for 2 h, followed by incubation with secondary antibodies including Cy3-conjugated anti-rabbit immunoglobulin G (IgG) and fluorescein isothiocyanate (FITC)-conjugated anti-mouse IgG antibodies (S4 Table). The sections were counterstained with 4′,6-diamidino-2-phenylindole (DAPI, Sigma-Aldrich, St. Louis, Missouri, USA). Images were viewed and captured by a Nikon DXM1200 fluorescence microscope (Nikon, Tokyo, Japan) attached to a computerized imaging system. The positive areas were analyzed at ×400 magnification, in 5 random fields, with the Image-Pro Plus Version 6.0 software (Media Cybernetics).

### ELISA for cytokines

The concentrations of sTNF-α in the heart homogenates, serum, and supernatants of cell culture were detected by enzyme-linked immunosorbent assay (ELISA), according to the manufacturer’s instructions (BioLegend, San Diego, California, USA).

### Knockdown of myocardial TNFR1 or TNFR2 in vivo

Type 9 rAAVs containing the cardiac troponin T (cTnT) promoter driving the expression of shTNFR1 (ctgcagGGAAGGAGTTCATGCGTTTcttcctgtcagaAAACGCATGAACTCCTTCCttttggatcc), shTNFR2 (ctgcagGAACCAGTTTCGTACATGTcttcctgtcagaACATGTACGAAACTGGTTCttttggatcc), or green fluorescent protein (GFP) were constructed and cotransfected with pHelper and pXX9 plasmids in human embryonic kidney 293T cells as described previously [43]. Male BALB/c mice were injected with rAAV-shTNFR1 or rAAV-shTNFR2 via the tail vein 1 × 10^11^ virion particles in 100 μl of saline solution. rAAV-GFP was used as a control. After 2 weeks, infected mice were subjected to either sham operation or TAC for 14 days.

### Quantitative real-time PCR

Total RNA was isolated from LV tissues, primary cardiomyocytes, or H9C2 cells by TRIzol according to the manufacturer's instructions (TransGen Biotech, Beijing, China). cDNA was reverse-transcribed from 1-μg RNA with the EasyScript 2-Step real-time PCR (RT-PCR) SuperMix (TransGen Biotech, Beijing, China). Relative levels of mRNA transcripts for atrial natriuretic peptide (*ANP*), brain natriuretic peptide (*BNP*), *TNF-α*, interleukin (*IL*)*-1β*, *IL-6*, *IL-10*, *TNFR1*, *TNFR2*, and *TACE* were quantified by RT-PCR with the use of TransStart Green qPCR SuperMix UDG kit (TransGen Biotech). Glyceraldehyde 3-phosphate dehydrogenase (GAPDH) transcripts were used to normalize the expression levels of the target genes by the ΔΔCt method. Primer pairs used for RT-PCR are listed in S5 Table.

### Isolation of adult cardiomyocytes from TNFR-KO mice

Adult cardiomyocytes were isolated as previously described [44]. Briefly, the heart of WT, TNFR1-/- and TNFR2-/-mice (8 weeks) were isolated and perfused with Ca2+-free bicarbonate buffer using a Langendorff system at a constant pressure (100 cm H_2_O) at 37°C for 3 min. Then, collagenases B (0.5 mg/ml), collagenases D (0.5 mg/ml), and protease XIV (0.02 mg/ml) were added to the perfusion solution for the enzymatic digestion. Cardiomyocytes were isolated and plated on laminin-coated dishes (1 × 10^4^ cells/cm^2^). The purity of cardiomyocytes was >95%.

### Cell culture and siRNA transfection

Embryonic rat heart-derived H9C2 cells (Cell Bank, Chinese Academy of Sciences, Shanghai, China) were maintained in Dulbecco’s modified Eagle’s medium (Invitrogen, Carlsbad, California, USA) supplemented with 10% fetal bovine serum (Invitrogen Gibco, Carlsbad, California, USA). H9C2 cells were seeded in 6-well culture dishes and transfected for 24 h with small interfering RNA (siRNA) against TNFR1 (sense: 5′-GUGAAGGAAUUGUUACUAAdTdT-3′, antisense: 3′-dTdTCACUUCCUUAACAAUGAUU-5′) or TNFR2 (sense: 5′-CCAAGGACAAUCUACGUAUdTdT-3′, antisense: 3′-dTdTGGUUCCUG UUAGAUGCAUA-5′) (Guangzhou RiboBio, Guangzhou, China) using Lipofectamine 2000 Transfection Reagent (Invitrogen), according to the manufacturer’s instructions.

### Exogenous tmTNF-ɑ and sTNF-α stimulation

NIH3T3 cells overexpressing tmTNF-α on the cell surface were fixed in 1% paraformaldehyde and used as the source of exogenous tmTNF-α as previously described [45]. To remove receptor-bound sTNF-α, cells were treated with acid glycine buffer (Gly-NaCl, pH 3.0) for 15 min after fixation [46].

At 24 h after transfection by siRNA, cells were treated with ISO (10 μMol/L) in the presence or absence of recombinant rat sTNF-α (20 ng/ml, Peprotech, Cranbury, New Jersey, USA) or tmTNF-ɑ (10:1 ration of the fixed NIH3T3 cells versus H9C2 cells) for additional 24 h.

### Immunocytofluorescence

H9C2 cells were washed with PBS and fixed in 4% paraformaldehyde for 10 min and then in 0.25% Triton-X100 for 10 min. After blocking with 5% bovine serum albumin (BSA) for 30 min, cells were incubated in Actin-Trakcer Green at 4°C overnight, according to the manufacturer’s instructions (Beyotime, Shanghai, China). Then, cells were visualized with DAPI (Sigma-Aldrich) for nuclear counter staining and subsequently observed under a Nikon DXM1200 fluorescence microscope. Measurement of cell area was performed using Image-Pro Plus 6.0 software (Media Cybernetics).

### Flow cytometry

After stimulation, cells were washed with cold PBS and incubated with a polyclonal antibody against TNF-α (LifeSpan BioSciences, Seattle, Washington state, USA) or TACE (ProSci, San Diego, California, USA) for 1 h at 4°C, followed by an FITC-labeled secondary antibody against rabbit IgG (Jackson Biotech, West Chester, Pennsylvania, USA) (S4 Table) for 1 h at 4°C. The expression of tmTNF-α on the cell surface was analyzed on an FACS Calibur 440E flow cytometer (Becton Dickinson, San Jose, California, USA).

### MTT assay

H9C2 cells as target cells were seeded at a density of 10^4^ cells per well (96-well plates) and transfected for 24 h with siRNA against TNFR1 or TNFR2. sTNF-α (20 ng/ml) or tmTNF-α on fixed NIH3T3 cells (at an effector/target ratio of 10:1) were added and incubated for additional 24 h. Vector-transfected NIH3T3 cells served as a control. Cell viability was determined by staining for 4 h with 30-mM glucose–PBS containing 0.5-mg/ml 3-(4,5-Dimethylthiazol-2-yl)-2,5-diphenyltetrazolium bromide (MTT) (Sigma, St. Louis, Missouri, USA), followed by lysis with 0.1-ml DMSO. The optical density (OD) value was measured at 570 nm on a microplate reader (Tecan, Groedig, Austria). Cytotoxicity or proliferation rate (%) was calculated by the following formula: Cytotoxicity (%) = (1 − OD_sample_/OD_control_) × 100%; Proliferation (%) = (OD_sample_/OD_control_) × 100%.

## Apoptosis

Cell apoptosis was detected by the FITC Annexin V Apoptosis Detection Kit (BD Pharmingen, USA) according to the manufacturer’s instructions.

Apoptosis in diseased heart tissue sections was detected using in situ FITC TUNEL Cell Apoptosis Detection Kit (Servicebio, Wuhan, Hubei, China) according to the manufacturer’s instructions. The sections were counterstained with DAPI.

### Proliferation

Cell proliferation was determined by the EdU assay kit (Guangzhou RiboBio) according to the manufacturer’s instructions.

### Mechanical stretch

siRNA-transfected H9C2 cells were seeded in silicon-based plates precoated with collagen and subjected to a 48-h mechanical stretch. Then, the H9C2 cells were collected for detection of *ANP*, *BNP*, and *TACE* transcription.

### Western immunoblotting

Western blotting was performed as described previously [47]. Briefly, total protein was extracted by using a lysis buffer, and 50 μg/lane was subjected to electrophoresis on SDS-polyacrylamide gels and transferred to polyvinylidene fluoride (PVDF) membranes (Millipore, Merck KGaA, Darmstadt, Germany). The membranes were blocked in 5% nonfat milk overnight and probed for 2 h with primary antibodies (Santa Cruz Biotechnology, Dallas, Texas, USA) including anti-TNFR1, anti-TNFR2, anti-TNF-α, anti-nuclear factor kappa B (NF-κB) p65 /p-p65, anti-AKT/p-AKT, and anti-GAPDH, followed by incubation with appropriate horseradish peroxidase (HRP)-conjugated secondary antibodies (S4 Table). Proteins were visualized using the enhanced chemiluminescence (ECL) system (Thermo Scientific) and the Kodak Image Station 4000 MM (Eastman Kodak, Rochester, New York). Bands were quantified by a calibrated imaging densitometer (GS-710; Bio-Rad, Hercules, California) and analyzed by "Quantity One" software (Bio-Rad).

### Statistical analysis

Data were statistically analyzed by 1-way or 2-way analysis of variance followed by Tukey post hoc tests using GraphPad Prism 6.0 software (San Diego, California, USA). Differences with *P* values of less than 0.05 were considered statistically significant.

## Supporting information

S1 Fig Aortic trans-TAC pressure gradients, survival, and fibrosis at 2 weeks after TAC. WT, TNFR1^-/-^, and TNFR2^-/-^ mice were subjected to pressure overload for 2 weeks by TAC, and sham-operated mice served as controls. (A and B) Representative pulsed wave Doppler images and quantitative data of velocity in aortic arch (*n* = 6 each group). (C) Peak trans-TAC pressure gradients calculated from Doppler velocities using the Bernoulli equation. (D) Kaplan–Meier survival curves for TAC (*n* = 20 each group, except TAC group in TNFR2 KO *n* = 18). (E) Fibrosis in LV tissues detected by Sirius red staining and quantitative data (*n* = 5, each group). **P < 0.01, ***P < 0.001 versus corresponding sham. See individual data at S1 Data.
(TIF)

S2 Fig Expression of TNFR1 and TNFR2 in cardiomyocytes after TAC and shRNA KD efficiency. WT, TNFR1^-/-^, and TNFR2^-/-^ mice were subjected to pressure overload for 2 weeks by TAC, and sham-operated mice served as controls. (A and B) Representative images of fluorescence co-immunostaining for troponin T (green) and TNFR1 or TNFR2 (red) on myocardial sections (400×) and quantitative data (*n* = 5, each group). (C and D) Quantitative data of indirect fluorescence costaining for troponin T and tmTNF-α (in Fig 2D) or TACE (in Fig 2G) on myocardial sections (*n* = 5, each group). (E and F) Western blot analysis of TNFR1 and TNR2 expression in primary myocardial cells isolated from WT mice at 2 weeks after intravenous injection with troponin T promoter containing rAAV-shTNFR1, rAAV-shTNFR2, or rAAV-GFP (1 × 10^11^ virion particles), and quantitative data. (G) Quantitative data of fluorescence immunostaining for TACE and troponin T on myocardial sections (in Fig 2N) (*n* = 5, each group). *P < 0.05, **P < 0.01, ***P < 0.001 versus sham (A-D and G) or rAAV-GFP (E and F). See individual data at S1 Data and underlying raw images at S1 Raw Images.
(TIF)

S3 Fig TNFR1 and TNFR2 differentially modulated cardiac hypertrophy and inflammation at 4 weeks after TAC. WT, TNFR1^-/-^, and TNFR2^-/-^ mice were subjected to pressure overload for 2 weeks by TAC, and sham-operated mice served as controls (*n* = 6 per group). Quantitative RT-PCR analysis of *ANP* (A), *BNP* (B), *IL-1β* (C), *IL-6* (D), *IL-10* (E), and *TNF-α* (F) in myocardial tissues. (G and H) sTNF-α concentrations in serum and heart homogenates detected by ELISA. (I) Representative western blots of tmTNF-α in myocardial tissues and quantitative data. (J and K) Representative images of indirect fluorescence costaining for troponin T and tmTNF-α or TACE on myocardial sections (400×) and quantitative data (*n* = 5 each group). (L) Representative images of fibrosis in myocardial tissues detected by Sirius red staining and quantitative data (*n* = 5, each group). *P < 0.05, **P < 0.01, ***P < 0.001 versus sham. Individual data can be found at S1 Data and underlying raw images at S1 Raw Images.
(TIF)

S4 Fig KD efficiency of siRNA for TNFR1 or TNFR2 and the effect of TNFR KD/KO on ISO-induced TNF-α transcription. H9C2 cells were transfected with control, TNFR1, or TNFR2 siRNA for indicated time points. (A and C) Quantitative RT-PCR analysis of *TNFR1* and *TNFR2*. (B and D) Representative images and quantitative data of western blots for TNFR1 and TNFR2 in H9C2 cells. (E and F) Quantitative RT-PCR analysis of *TNF-α* in H9C2 cells transfected with control, TNFR1, or TNFR2 siRNA or in primary myocardiocytes from WT, TNFR1-KO, or TNFR2-KO mice stimulated with ISO (10 μM) for 24 h. All the quantitative data represent as mean ± SE of at least 3 independent experiments. *P < 0.05, **P < 0.01, ***P < 0.001 versus siCtrl for A-D or vehicle for E and F. Individual data are included in S1 Data and underlying raw images in S1 Raw Images.
(TIF)

S5 Fig tmTNF-α induced proliferation but sTNF-α mediated apoptosis in cardiomyocytes. (A and B) WT, TNFR1^-/-^, and TNFR2^-/-^ mice were subjected to pressure overload for 2 and 4 weeks by TAC, and sham-operated mice served as controls. Representative images of apoptosis detected by TUNEL staining in myocardial tissues and quantitative data (*n* = 5, each group). (C–F) H9C2 cells transfected for 24 h with siRNA against TNFR1 or TNFR2 were cultured for additional 24 h with sTNF-α (20 ng/ml) or tmTNF-α on fixed NIH3T3 cells (at an effector/target ratio of 10:1). Vector-transfected NIH3T3 cells served as a control. (C) Representative cytograms of sTNF-α-induced apoptosis in H9C2 cells detected by Anexin V and quantitative data. (D) sTNF-α-mediated cytotoxicity toward H9C2 cells determined by MTT assay. (E) Representative images of EdU staining and quantitative data. (F) tmTNF-α-induced proliferation of H9C2 cells detected by MTT assay. The quantitative data of C–F represent as mean ± SE of 3 to 5 independent experiments. *P < 0.05, **P < 0.01, ***P < 0.001 versus shame for A and B or control for C and D or vector for E and F. Find individual data in S1 Data.
(TIF)

S6 Fig TNFR KD regulated stretch-induced TACE expression. H9C2 cells were exposed to stretch for 48 h. (A–C) Quantitative RT-PCR analysis of *ANP*, *BNP*, and *TACE*. (D) TACE expression on the cell surface of H9C2 cells detected by flow cytometry and quantitative data. (E) H9C2 cells transfected for 24 h with siRNA against TNFR1 or TNFR2 were exposed to stretch for 48 h. Quantitative RT-PCR analysis of *TACE*. All quantitative data represent as mean ± SE of 3 independent experiments. *P < 0.05, **P < 0.01, ***P < 0.001 versus control. Individual data can be found in S1 Data.
(TIF)

S1 Table Echocardiographic and hemodynamic analysis in WT, TNFR1,-/- and TNFR2-/- mice at 2 weeks after sham or TAC operation. (DOCX)

S2 Table Echocardiographic and hemodynamic analysis in mice injected with rAAV-GFP, rAAV-TNFR1 shRNA, or rAAV-TNFR2 shRNA at 2 weeks after sham or TAC operation. (DOCX)

S3 Table Echocardiographic and hemodynamic analysis in WT, TNFR1-/-, and TNFR2-/- mice at 4 weeks after sham or TAC operation. (DOCX)

S4 Table Antibodies used in this study. (DOCX)

S5 Table Primer sequences for quantitative RT-PCR. (DOCX)

S1 Data Data underlying Figs 1–7 and S1–S6 Figs. (XLSX)

S1 Raw Images Original gel and images contained in this manuscript. (PDF)

## Abbreviations

ADAM: adisintegrin and metalloproteinase
ANP: atrial natriuretic peptide
BNP: brain natriuretic peptide
BSA: bovine serum albumin
cTnT: cardiac troponin T
DAPI: 4′,6-diamidino-2-phenylindole
dP/dt_max_: peak instantaneous rate of left ventricular pressure increase
dP/dt_min_: peak instantaneous rate of left ventricular pressure increase decline
ECL: enhanced chemiluminescence
EF: ejection fraction
ELISA: enzyme-linked immunosorbent assay
FITC: fluorescein isothiocyanate
FS: fractional shortening
GAPDH: glyceraldehyde 3-phosphate dehydrogenase
GFP: green fluorescent protein
HW/BW: heart weight to body weight
HF: heart failure
HR: heart rate
HRP: horseradish peroxidase
IgG: immunoglobulin G
IL: interleukin
ISO: isoproterenol
KO: knockout
KD: knockdown
LV: left ventricle
LVAW,d: LV anterior wall thickness at end-diastole
LVEDP: LV end-diastolic pressure
LVESP: LV end-systolic pressure
LVID,d: LV internal diameter at end-diastole
LVPW,d: LV posterior wall thickness at end-diastole
MMP: matrix metalloprotease
MTT: 3-(4,5-Dimethylthiazol-2-yl)-2,5-diphenyltetrazolium bromide
NF: nuclear factor
NF-κB: nuclear factor kappa B
OD: optical density
PVDF: polyvinylidene fluoride
RT-PCR: real-time PCR
siRNA: small interfering RNA
sTNF-α: soluble TNF-α
TAC: transverse aortic constriction
TACE: TNF-α-converting enzyme
TAPI-1: TNF-α Protease Inhibitor-1
tmTNF-α: transmembrane TNF-α
TNF-α: tumor necrosis factor-alpha
TNFR: TNF receptor
WT: wild-type

## References

[1] Adamopoulos S, Parissis JT, Kremastinos DT. A glossary of circulating cytokines in chronic heart failure. Eur J Heart Fail. 2001;3(5):517–26. 10.1016/s1388-9842(01)00156-8 . 11595599

[2] Mann DL. Inflammatory mediators and the failing heart: past, present, and the foreseeable future. Circ Res. 2002;91(11):988–98. 10.1161/01.res.0000043825.01705.1b . 12456484

[3] Grandel U, Fink L, Blum A, Heep M, Buerke M, Kraemer HJ, et al Endotoxin-induced myocardial tumor necrosis factor-alpha synthesis depresses contractility of isolated rat hearts: evidence for a role of sphingosine and cyclooxygenase-2-derived thromboxane production. Circulation. 2000;102(22):2758–64. 10.1161/01.cir.102.22.2758 . 11094044

[4] Yokoyama T, Nakano M, Bednarczyk JL, McIntyre BW, Entman M, Mann DL. Tumor necrosis factor-alpha provokes a hypertrophic growth response in adult cardiac myocytes. Circulation. 1997;95(5):1247–52. 10.1161/01.cir.95.5.1247 . 9054856

[5] Bradham WS, Bozkurt B, Gunasinghe H, Mann D, Spinale FG. Tumor necrosis factor-alpha and myocardial remodeling in progression of heart failure: a current perspective. Cardiovasc Res. 2002;53(4):822–30. 10.1016/s0008-6363(01)00503-x . 11922892

[6] Kubota T, McTiernan CF, Frye CS, Slawson SE, Lemster BH, Koretsky AP, et al Dilated cardiomyopathy in transgenic mice with cardiac-specific overexpression of tumor necrosis factor-alpha. Circ Res. 1997;81(4):627–35. 10.1161/01.res.81.4.627 . 9314845

[7] Stamm C, Friehs I, Cowan DB, Moran AM, Cao-Danh H, Duebener LF, et al Inhibition of tumor necrosis factor-alpha improves postischemic recovery of hypertrophied hearts. Circulation. 2001;104(12 Suppl 1):I350–5. 10.1161/hc37t1.094851 . 11568081

[8] Balakumar P, Singh M. Anti-tumour necrosis factor-alpha therapy in heart failure: future directions. Basic Clin Pharmacol Toxicol. 2006;99(6):391–7. 10.1111/j.1742-7843.2006.pto_508.x . 17169118

[9] Mann DL, McMurray JJ, Packer M, Swedberg K, Borer JS, Colucci WS, et al Targeted anticytokine therapy in patients with chronic heart failure: results of the Randomized Etanercept Worldwide Evaluation (RENEWAL). Circulation. 2004;109(13):1594–602. 10.1161/01.CIR.0000124490.27666.B2 . 15023878

[10] Chung ES, Packer M, Lo KH, Fasanmade AA, Willerson JT, Anti TNFTACHFI. Randomized, double-blind, placebo-controlled, pilot trial of infliximab, a chimeric monoclonal antibody to tumor necrosis factor-alpha, in patients with moderate-to-severe heart failure: results of the anti-TNF Therapy Against Congestive Heart Failure (ATTACH) trial. Circulation. 2003;107(25):3133–40. 10.1161/01.CIR.0000077913.60364.D2 . 12796126

[11] Javed Q, Murtaza I. Therapeutic potential of tumour necrosis factor-alpha antagonists in patients with chronic heart failure. Heart Lung Circ. 2013;22(5):323–7. 10.1016/j.hlc.2012.12.002 . 23337264

[12] Li Q, Li L, Shi W, Jiang X, Xu Y, Gong F, et al Mechanism of action differences in the antitumor effects of transmembrane and secretory tumor necrosis factor-alpha in vitro and in vivo. Cancer Immunol Immunother. 2006;55(12):1470–9. 10.1007/s00262-006-0150-x . 16555058 PMC11030923

[13] Zhou W, Yang P, Liu L, Zheng S, Zeng Q, Liang H, et al Transmembrane tumor necrosis factor-alpha sensitizes adipocytes to insulin. Mol Cell Endocrinol. 2015;406:78–86. 10.1016/j.mce.2015.02.023 . 25725372

[14] Satoh M, Nakamura M, Saitoh H, Satoh H, Maesawa C, Segawa I, et al Tumor necrosis factor-alpha-converting enzyme and tumor necrosis factor-alpha in human dilated cardiomyopathy. Circulation. 1999;99(25):3260–5. 10.1161/01.cir.99.25.3260 . 10385500

[15] Dibbs ZI, Diwan A, Nemoto S, DeFreitas G, Abdellatif M, Carabello BA, et al Targeted overexpression of transmembrane tumor necrosis factor provokes a concentric cardiac hypertrophic phenotype. Circulation. 2003;108(8):1002–8. 10.1161/01.CIR.0000085203.46621.F4 . 12912811

[16] Diwan A, Dibbs Z, Nemoto S, DeFreitas G, Carabello BA, Sivasubramanian N, et al Targeted overexpression of noncleavable and secreted forms of tumor necrosis factor provokes disparate cardiac phenotypes. Circulation. 2004;109(2):262–8. 10.1161/01.CIR.0000109642.27985.FA . 14699008

[17] Naude PJ, den Boer JA, Luiten PG, Eisel UL. Tumor necrosis factor receptor cross-talk. FEBS J. 2011;278(6):888–98. 10.1111/j.1742-4658.2011.08017.x . 21232019

[18] Hamid T, Gu Y, Ortines RV, Bhattacharya C, Wang G, Xuan YT, et al Divergent tumor necrosis factor receptor-related remodeling responses in heart failure: role of nuclear factor-kappaB and inflammatory activation. Circulation. 2009;119(10):1386–97. 10.1161/CIRCULATIONAHA.108.802918 . Pubmed Central PMCID: 2730645. 19255345 PMC2730645

[19] Blessing E, Bea F, Kuo CC, Campbell LA, Chesebro B, Rosenfeld ME. Lesion progression and plaque composition are not altered in older apoE-/- mice lacking tumor necrosis factor-alpha receptor p55. Atherosclerosis. 2004;176(2):227–32. 10.1016/j.atherosclerosis.2004.05.033 . 15380444

[20] Flaherty MP, Guo Y, Tiwari S, Rezazadeh A, Hunt G, Sanganalmath SK, et al The role of TNF-alpha receptors p55 and p75 in acute myocardial ischemia/reperfusion injury and late preconditioning. J Mol Cell Cardiol. 2008;45(6):735–41. 10.1016/j.yjmcc.2008.08.014 . Pubmed Central PMCID: 2645001. 18824172 PMC2645001

[21] Kurrelmeyer KM, Michael LH, Baumgarten G, Taffet GE, Peschon JJ, Sivasubramanian N, et al Endogenous tumor necrosis factor protects the adult cardiac myocyte against ischemic-induced apoptosis in a murine model of acute myocardial infarction. Proc Natl Acad Sci U S A. 2000;97(10):5456–61. 10.1073/pnas.070036297 . Pubmed Central PMCID: 25850. 10779546 PMC25850

[22] Zhang Y, Zhao J, Lau WB, Jiao LY, Liu B, Yuan Y, et al Tumor necrosis factor-alpha and lymphotoxin-alpha mediate myocardial ischemic injury via TNF receptor 1, but are cardioprotective when activating TNF receptor 2. PLoS ONE. 2013;8(5):e60227 10.1371/journal.pone.0060227 . Pubmed Central PMCID: 3660398. 23704873 PMC3660398

[23] Yu M, Zhou X, Niu L, Lin G, Huang J, Zhou W, et al Targeting transmembrane TNF-alpha suppresses breast cancer growth. Cancer Res. 2013;73(13):4061–74. 10.1158/0008-5472.CAN-12-3946 . 23794706

[24] Park D, Lee HS, Kang JH, Kim SM, Gong JR, Cho KH. Attractor landscape analysis of the cardiac signaling network reveals mechanism-based therapeutic strategies for heart failure. J Mol Cell Biol. 2018;10(3):180–94. 10.1093/jmcb/mjy019 . 29579284

[25] Jarrah AA, Schwarskopf M, Wang ER, LaRocca T, Dhume A, Zhang S, et al SDF-1 induces TNF-mediated apoptosis in cardiac myocytes. Apoptosis. 2018;23(1):79–91. 10.1007/s10495-017-1438-3 . Pubmed Central PMCID: 6019273. 29236198 PMC6019273

[26] Wang QL, Zhao L, Feng N, Zhou P, Wu Q, Fan R, et al Lacidipine attenuates TNF-alpha-induced cardiomyocyte apoptosis. Cytokine. 2015;71(1):60–5. 10.1016/j.cyto.2014.08.004 . 25226445

[27] Bzowska M, Jura N, Lassak A, Black RA, Bereta J. Tumour necrosis factor-alpha stimulates expression of TNF-alpha converting enzyme in endothelial cells. Eur J Biochem. 2004;271(13):2808–20. 10.1111/j.1432-1033.2004.04215.x . 15206946

[28] Higuchi Y, McTiernan CF, Frye CB, McGowan BS, Chan TO, Feldman AM. Tumor necrosis factor receptors 1 and 2 differentially regulate survival, cardiac dysfunction, and remodeling in transgenic mice with tumor necrosis factor-alpha-induced cardiomyopathy. Circulation. 2004;109(15):1892–7. 10.1161/01.CIR.0000124227.00670.AB . 15051641

[29] Duerrschmid C, Crawford JR, Reineke E, Taffet GE, Trial J, Entman ML, et al TNF receptor 1 signaling is critically involved in mediating angiotensin-II-induced cardiac fibrosis. J Mol Cell Cardiol. 2013;57:59–67. 10.1016/j.yjmcc.2013.01.006 . Pubmed Central PMCID: 3593947. 23337087 PMC3593947

[30] Fedak PW, Smookler DS, Kassiri Z, Ohno N, Leco KJ, Verma S, et al TIMP-3 deficiency leads to dilated cardiomyopathy. Circulation. 2004;110(16):2401–9. 10.1161/01.CIR.0000134959.83967.2D . 15262835

[31] Satoh M, Iwasaka J, Nakamura M, Akatsu T, Shimoda Y, Hiramori K. Increased expression of tumor necrosis factor-alpha converting enzyme and tumor necrosis factor-alpha in peripheral blood mononuclear cells in patients with advanced congestive heart failure. Eur J Heart Fail. 2004;6(7):869–75. 10.1016/j.ejheart.2004.02.007 . 15556048

[32] Jubair S, Li J, Dehlin HM, Manteufel EJ, Goldspink PH, Levick SP, et al Substance P induces cardioprotection in ischemia-reperfusion via activation of AKT. Am J Physiol Heart Circ Physiol. 2015;309(4):H676–84. 10.1152/ajpheart.00200.2015 . Pubmed Central PMCID: 4537946. 26071541 PMC4537946

[33] Moc C, Taylor AE, Chesini GP, Zambrano CM, Barlow MS, Zhang X, et al Physiological activation of Akt by PHLPP1 deletion protects against pathological hypertrophy. Cardiovasc Res. 2015;105(2):160–70. 10.1093/cvr/cvu243 . Pubmed Central PMCID: 4303795. 25411382 PMC4303795

[34] Rota M, Boni A, Urbanek K, Padin-Iruegas ME, Kajstura TJ, Fiore G, et al Nuclear targeting of Akt enhances ventricular function and myocyte contractility. Circ Res. 2005;97(12):1332–41. 10.1161/01.RES.0000196568.11624.ae . 16293788

[35] Su F, Zhao L, Zhang S, Wang J, Chen N, Gong Q, et al Cardioprotection by PI3K-mediated signaling is required for anti-arrhythmia and myocardial repair in response to ischemic preconditioning in infarcted pig hearts. Lab Investig. 2015;95(8):860–71. 10.1038/labinvest.2015.64 . 26006021

[36] Freund C, Schmidt-Ullrich R, Baurand A, Dunger S, Schneider W, Loser P, et al Requirement of nuclear factor-kappaB in angiotensin II- and isoproterenol-induced cardiac hypertrophy in vivo. Circulation. 2005;111(18):2319–25. 10.1161/01.CIR.0000164237.58200.5A . 15870116

[37] Gupta S, Young D, Sen S. Inhibition of NF-kappaB induces regression of cardiac hypertrophy, independent of blood pressure control, in spontaneously hypertensive rats. Am J Physiol Heart Circ Physiol. 2005;289(1):H20–9. 10.1152/ajpheart.00082.2005 . 15749748

[38] Young D, Popovic ZB, Jones WK, Gupta S. Blockade of NF-kappaB using IkappaB alpha dominant-negative mice ameliorates cardiac hypertrophy in myotrophin-overexpressed transgenic mice. J Mol Biol. 2008;381(3):559–68. 10.1016/j.jmb.2008.05.076 . Pubmed Central PMCID: 2688722. 18620706 PMC2688722

[39] Lin Z, Zhou P, von Gise A, Gu F, Ma Q, Chen J, et al Pi3kcb links Hippo-YAP and PI3K-AKT signaling pathways to promote cardiomyocyte proliferation and survival. Circ Res. 2015;116(1):35–45. 10.1161/CIRCRESAHA.115.304457 . Pubmed Central PMCID: 4282610. 25249570 PMC4282610

[40] Kaymakcalan Z, Sakorafas P, Bose S, Scesney S, Xiong L, Hanzatian DK, et al Comparisons of affinities, avidities, and complement activation of adalimumab, infliximab, and etanercept in binding to soluble and membrane tumor necrosis factor. Clin Immunol. 2009;131(2):308–16. 10.1016/j.clim.2009.01.002 . 19188093

[41] Zhao X, Mohaupt M, Jiang J, Liu S, Li B, Qin Z. Tumor necrosis factor receptor 2-mediated tumor suppression is nitric oxide dependent and involves angiostasis. Cancer Res. 2007;67(9):4443–50. 10.1158/0008-5472.CAN-07-0185 . 17483359

[42] Zhou L, Miao K, Yin B, Li H, Fan J, Zhu Y, et al Cardioprotective Role of Myeloid-Derived Suppressor Cells in Heart Failure. Circulation. 2018;138(2):181–97. 10.1161/CIRCULATIONAHA.117.030811 . 29437117

[43] Li H, Zhang X, Wang F, Zhou L, Yin Z, Fan J, et al MicroRNA-21 Lowers Blood Pressure in Spontaneous Hypertensive Rats by Upregulating Mitochondrial Translation. Circulation. 2016;134(10):734–51. 10.1161/CIRCULATIONAHA.116.023926 . Pubmed Central PMCID: 5515592. 27542393 PMC5515592

[44] Wang B, Nie J, Wu L, Hu Y, Wen Z, Dong L, et al AMPKalpha2 Protects Against the Development of Heart Failure by Enhancing Mitophagy via PINK1 Phosphorylation. Circ Res. 2018;122(5):712–29. 10.1161/CIRCRESAHA.117.312317 . Pubmed Central PMCID: 5834386. 29284690 PMC5834386

[45] Chen H, Xiao L, Zhang H, Liu N, Liu T, Liu L, et al The involvement of beta-actin in the signaling of transmembrane TNF-alpha-mediated cytotoxicity. J Leukoc Biol. 2011;89(6):917–26. 10.1189/jlb.1209812 . 21402772

[46] Zhang H, Yan D, Shi X, Liang H, Pang Y, Qin N, et al Transmembrane TNF-alpha mediates "forward" and "reverse" signaling, inducing cell death or survival via the NF-kappaB pathway in Raji Burkitt lymphoma cells. J Leukoc Biol. 2008;84(3):789–97. 10.1189/jlb.0208078 . Pubmed Central PMCID: 2516903. 18550789 PMC2516903

[47] Zhao C, Wang P, Xiao X, Chao J, Chao L, Wang DW, et al Gene therapy with human tissue kallikrein reduces hypertension and hyperinsulinemia in fructose-induced hypertensive rats. Hypertension. 2003;42(5):1026–33. 10.1161/01.HYP.0000097603.55404.35 . 14568997

