# Supplementary figures and images for: Transmembrane tumor necrosis factor alpha attenuates pressure-overload cardiac hypertrophy via tumor necrosis factor receptor 2
Source: PLoS Biol. 2020 Dec 3;18(12):e3000967. doi: 10.1371/journal.pbio.3000967 (PMC7714153; doi:10.1371/journal.pbio.3000967)

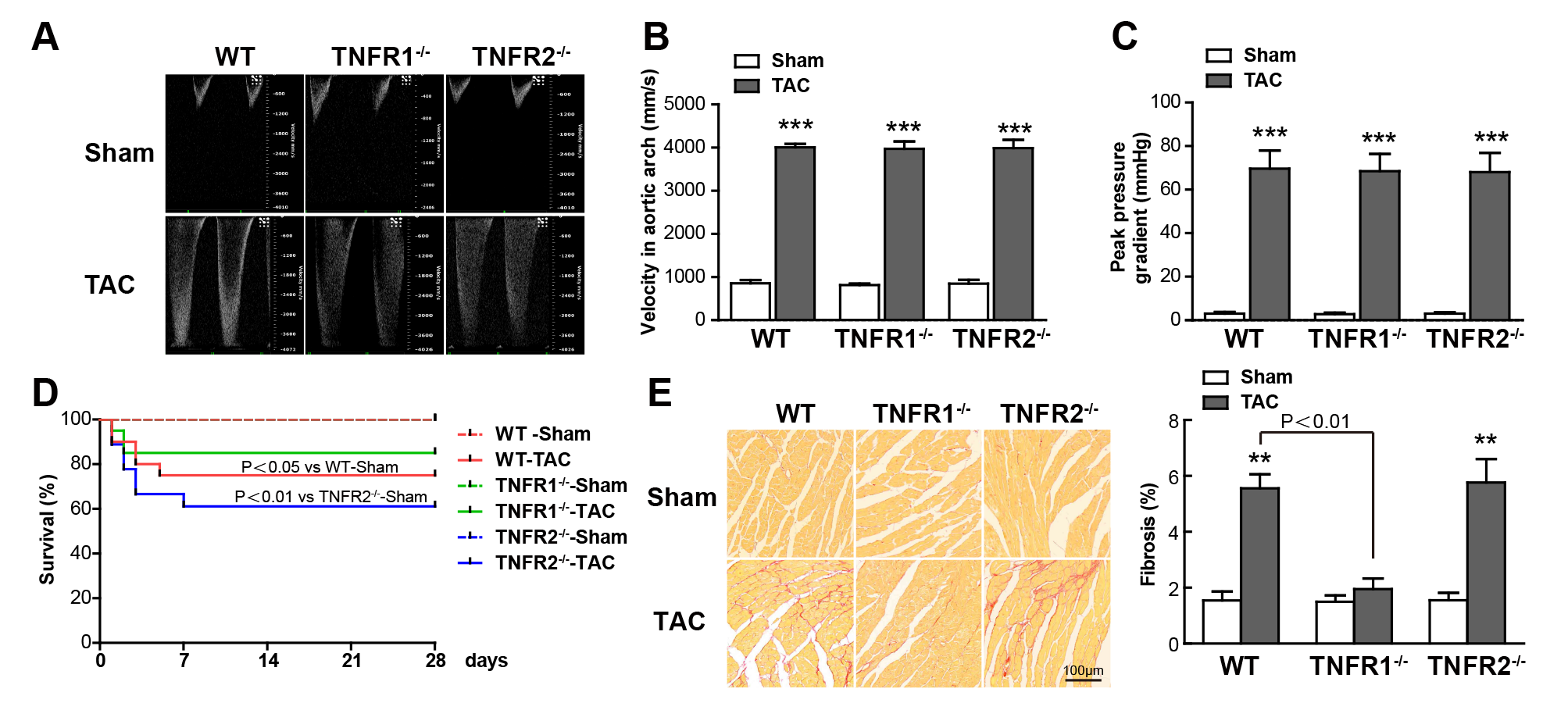

Supplement: S1 Fig — WT, TNFR1-/-, and TNFR2-/- mice were subjected to pressure overload for 2 weeks by TAC, and sham-operated mice served as controls. (A and B) Representative pulsed wave Doppler images and quantitative data of velocity in aortic arch (n = 6 each group). (C) Peak trans-TAC pressure gradients calculated from Doppler velocities using the Bernoulli equation. (D) Kaplan–Meier survival curves for TAC (n = 20 each group, except TAC group in TNFR2 KO n = 18). (E) Fibrosis in LV tissues detected by Sirius red staining and quantitative data (n = 5, each group). **P < 0.01, ***P < 0.001 versus corresponding sham. See individual data at S1 Data. (TIF) [file pbio.3000967.s001.tif]

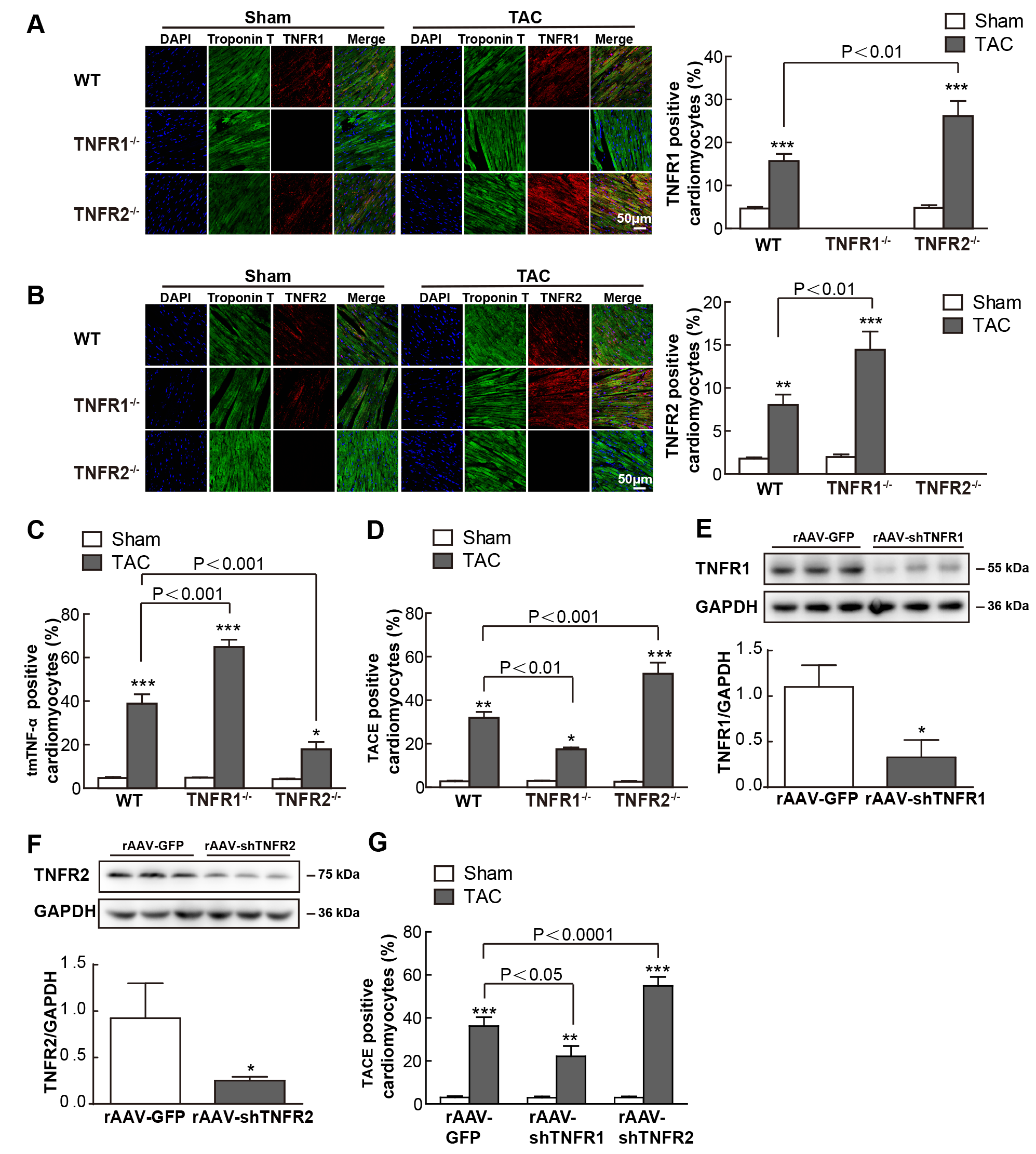

Supplement: S2 Fig — WT, TNFR1-/-, and TNFR2-/- mice were subjected to pressure overload for 2 weeks by TAC, and sham-operated mice served as controls. (A and B) Representative images of fluorescence co-immunostaining for troponin T (green) and TNFR1 or TNFR2 (red) on myocardial sections (400×) and quantitative data (n = 5, each group). (C and D) Quantitative data of indirect fluorescence costaining for troponin T and tmTNF-α (in Fig 2D) or TACE (in Fig 2G) on myocardial sections (n = 5, each group). (E and F) Western blot analysis of TNFR1 and TNR2 expression in primary myocardial cells isolated from WT mice at 2 weeks after intravenous injection with troponin T promoter containing rAAV-shTNFR1, rAAV-shTNFR2, or rAAV-GFP (1 × 10^11^ virion particles), and quantitative data. (G) Quantitative data of fluorescence immunostaining for TACE and troponin T on myocardial sections (in Fig 2N) (n = 5, each group). *P < 0.05, **P < 0.01, ***P < 0.001 versus sham (A-D and G) or rAAV-GFP (E and F). See individual data at S1 Data and underlying raw images at S1 Raw Images. (TIF) [file pbio.3000967.s002.tif]

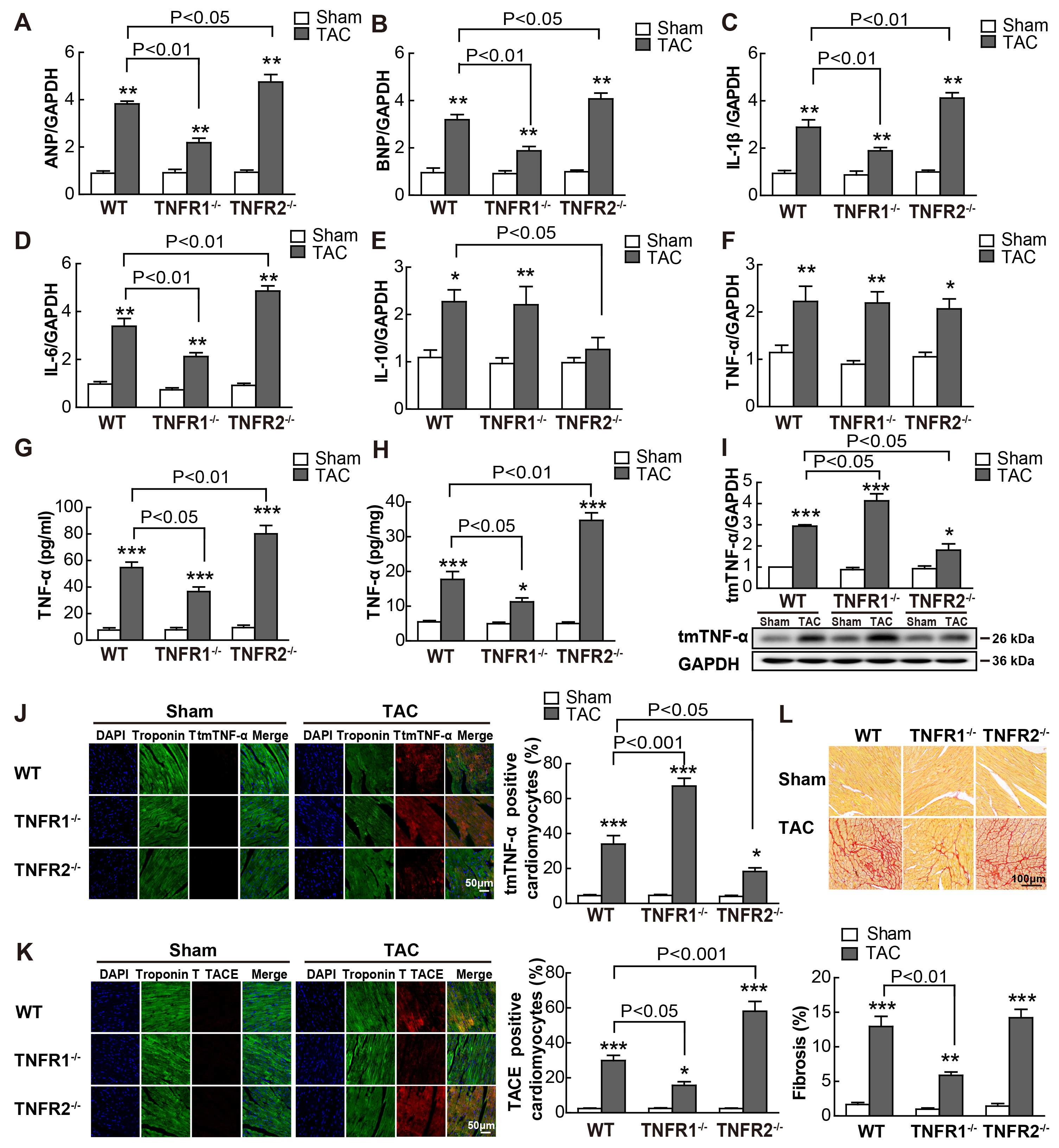

Supplement: S3 Fig — WT, TNFR1-/-, and TNFR2-/- mice were subjected to pressure overload for 2 weeks by TAC, and sham-operated mice served as controls (n = 6 per group). Quantitative RT-PCR analysis of ANP (A), BNP (B), IL-1β (C), IL-6 (D), IL-10 (E), and TNF-α (F) in myocardial tissues. (G and H) sTNF-α concentrations in serum and heart homogenates detected by ELISA. (I) Representative western blots of tmTNF-α in myocardial tissues and quantitative data. (J and K) Representative images of indirect fluorescence costaining for troponin T and tmTNF-α or TACE on myocardial sections (400×) and quantitative data (n = 5 each group). (L) Representative images of fibrosis in myocardial tissues detected by Sirius red staining and quantitative data (n = 5, each group). *P < 0.05, **P < 0.01, ***P < 0.001 versus sham. Individual data can be found at S1 Data and underlying raw images at S1 Raw Images. (TIF) [file pbio.3000967.s003.tif]

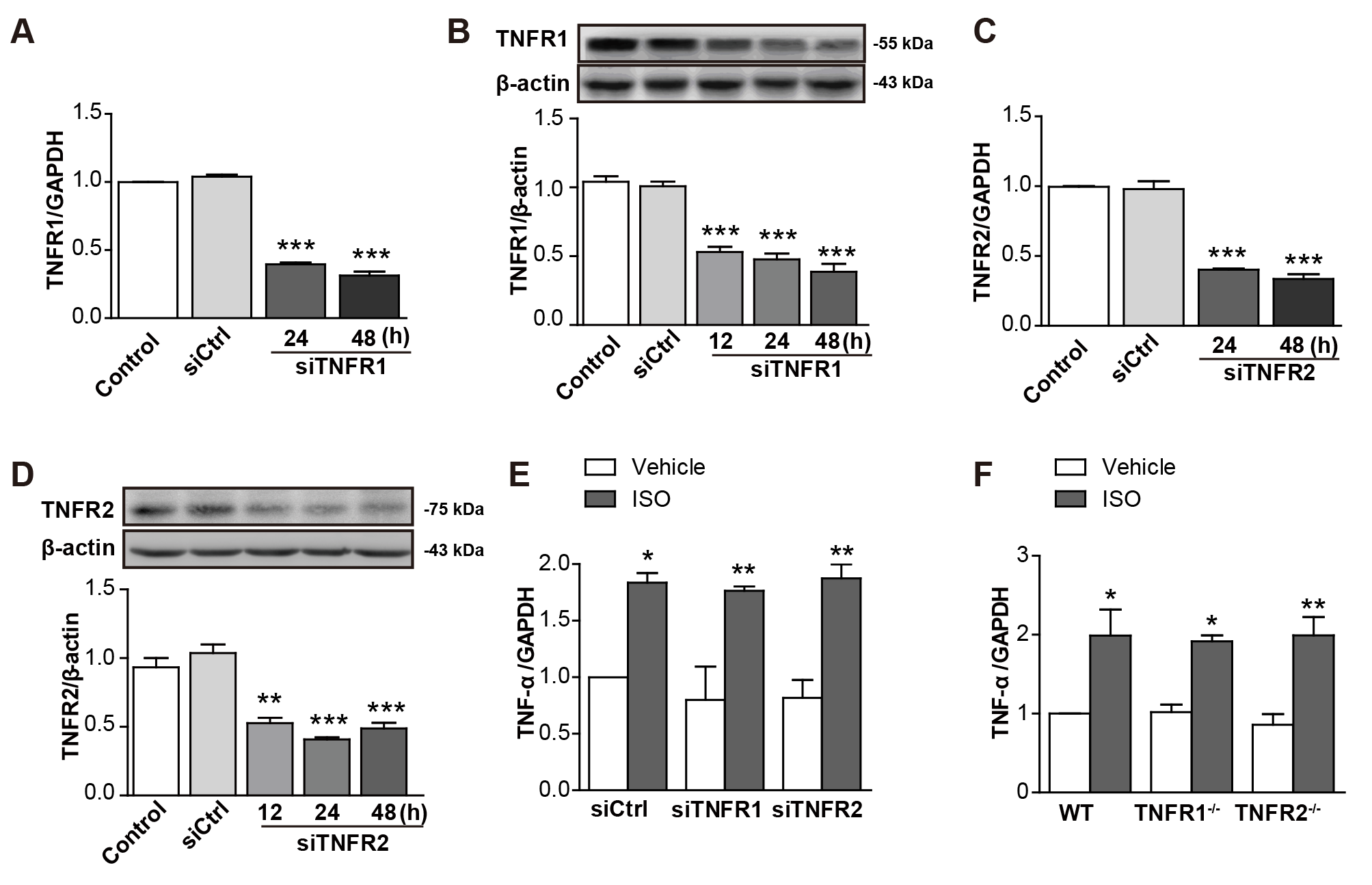

Supplement: S4 Fig — H9C2 cells were transfected with control, TNFR1, or TNFR2 siRNA for indicated time points. (A and C) Quantitative RT-PCR analysis of TNFR1 and TNFR2. (B and D) Representative images and quantitative data of western blots for TNFR1 and TNFR2 in H9C2 cells. (E and F) Quantitative RT-PCR analysis of TNF-α in H9C2 cells transfected with control, TNFR1, or TNFR2 siRNA or in primary myocardiocytes from WT, TNFR1-KO, or TNFR2-KO mice stimulated with ISO (10 μM) for 24 h. All the quantitative data represent as mean ± SE of at least 3 independent experiments. *P < 0.05, **P < 0.01, ***P < 0.001 versus siCtrl for A-D or vehicle for E and F. Individual data are included in S1 Data and underlying raw images in S1 Raw Images. (TIF) [file pbio.3000967.s004.tif]

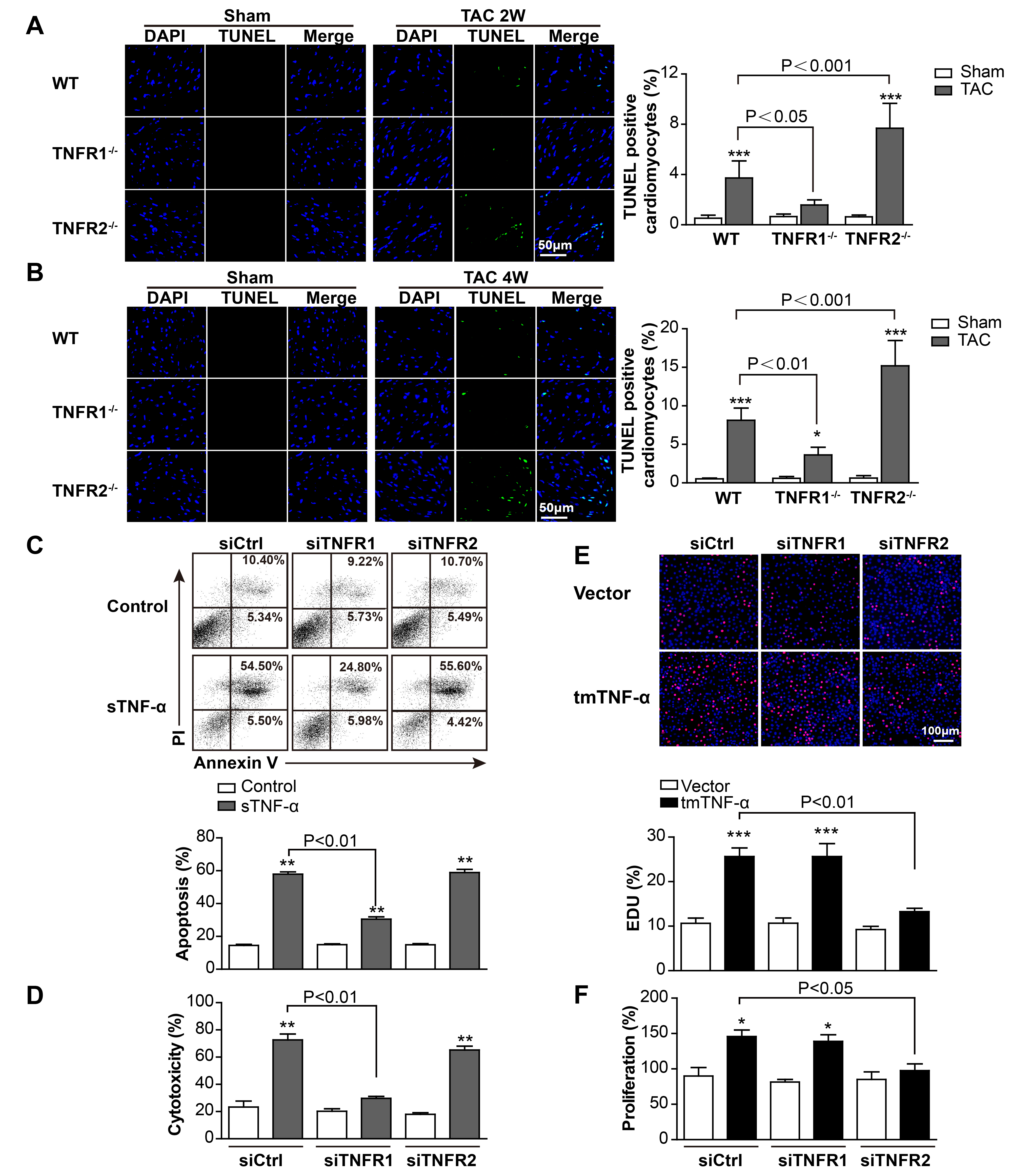

Supplement: S5 Fig — (A and B) WT, TNFR1-/-, and TNFR2-/- mice were subjected to pressure overload for 2 and 4 weeks by TAC, and sham-operated mice served as controls. Representative images of apoptosis detected by TUNEL staining in myocardial tissues and quantitative data (n = 5, each group). (C–F) H9C2 cells transfected for 24 h with siRNA against TNFR1 or TNFR2 were cultured for additional 24 h with sTNF-α (20 ng/ml) or tmTNF-α on fixed NIH3T3 cells (at an effector/target ratio of 10:1). Vector-transfected NIH3T3 cells served as a control. (C) Representative cytograms of sTNF-α-induced apoptosis in H9C2 cells detected by Anexin V and quantitative data. (D) sTNF-α-mediated cytotoxicity toward H9C2 cells determined by MTT assay. (E) Representative images of EdU staining and quantitative data. (F) tmTNF-α-induced proliferation of H9C2 cells detected by MTT assay. The quantitative data of C–F represent as mean ± SE of 3 to 5 independent experiments. *P < 0.05, **P < 0.01, ***P < 0.001 versus shame for A and B or control for C and D or vector for E and F. Find individual data in S1 Data. (TIF) [file pbio.3000967.s005.tif]

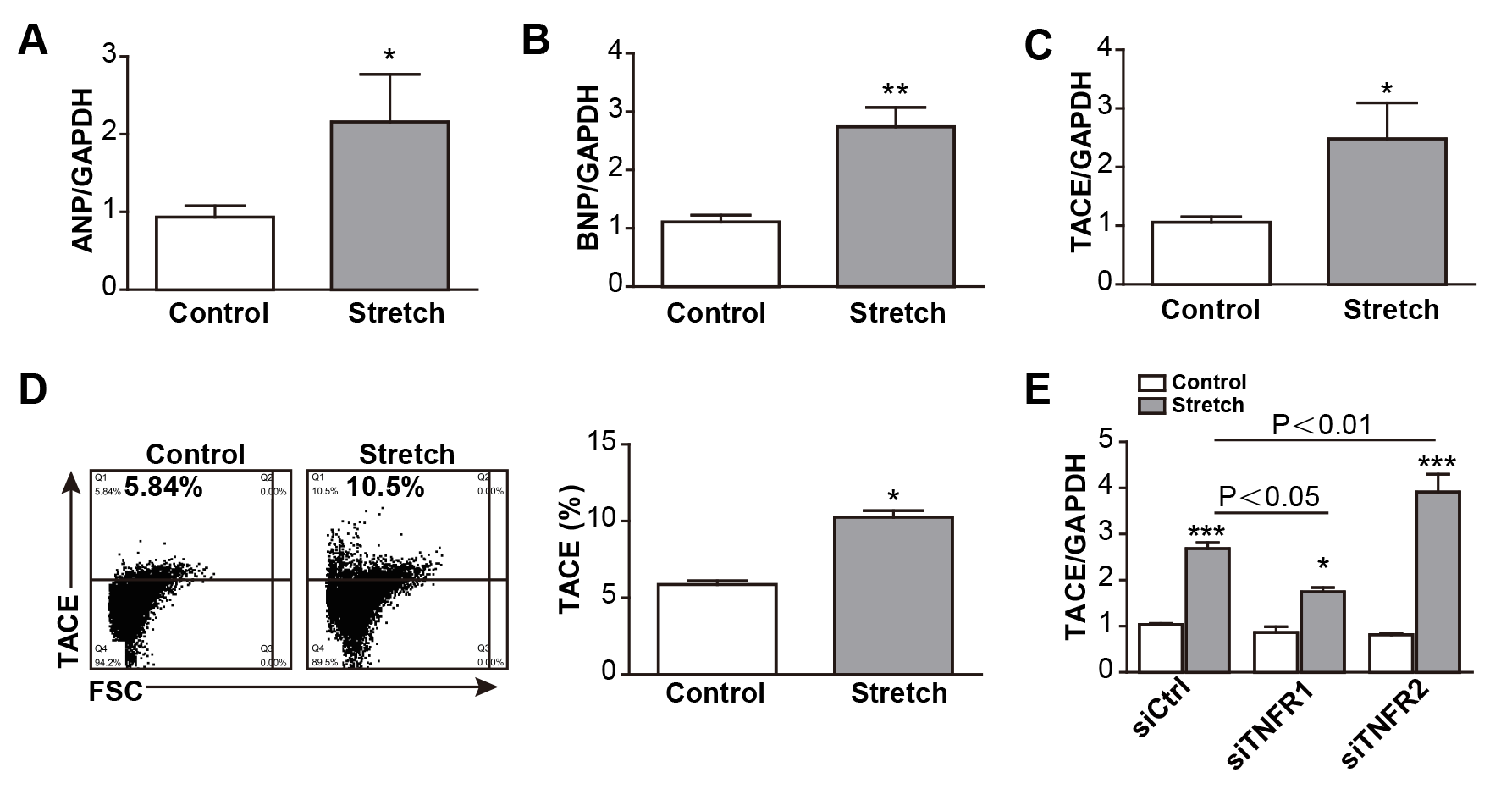

Supplement: S6 Fig — H9C2 cells were exposed to stretch for 48 h. (A–C) Quantitative RT-PCR analysis of ANP, BNP, and TACE. (D) TACE expression on the cell surface of H9C2 cells detected by flow cytometry and quantitative data. (E) H9C2 cells transfected for 24 h with siRNA against TNFR1 or TNFR2 were exposed to stretch for 48 h. Quantitative RT-PCR analysis of TACE. All quantitative data represent as mean ± SE of 3 independent experiments. *P < 0.05, **P < 0.01, ***P < 0.001 versus control. Individual data can be found in S1 Data. (TIF) [file pbio.3000967.s006.tif]
