# Supplementary material for: Transmembrane tumor necrosis factor alpha attenuates pressure-overload cardiac hypertrophy via tumor necrosis factor receptor 2
Source: PLoS Biol. 2020 Dec 3;18(12):e3000967. doi: 10.1371/journal.pbio.3000967 (PMC7714153; doi:10.1371/journal.pbio.3000967)
Supplement: S1 Table — (DOCX) [file pbio.3000967.s007.docx]

**S1 Table. Echocardiographic and hemodynamic analysis in WT, TNFR1^-/-^ and TNFR2^-/-^ mice at 2 weeks after sham or TAC operation**

|  | Sham | | | TAC | | |
| --- | --- | --- | --- | --- | --- | --- |
|  | WT | TNFR1^-/-^ | TNFR2^-/-^ | WT | TNFR1^-/-^ | TNFR2^-/-^ |
| BW (g) | 25.47±0.66 | 24.94±0.53 | 25.05±0.90 | 25.21±0.55 | 25.05±0.57 | 24.37±0.84 |
| **Echocardiography** |  |  |  |  |  |  |
| HR (b.p.m) | 451.4±15.2 | 458.5±13.2 | 461.4±12.4 | 447.4±10.3 | 437.5±11.5 | 435.0±12.7 |
| LV mass（mg） | 73.05±2.48 | 72.71±3.21 | 70.42±2.51 | 103.41±2.59 | 87.12±2.41 | 118.57±2.96 |
| LV mass/BW（mg/g） | 2.89±0.16 | 2.93±0.19 | 2.82±0.11 | 4.11±0.10** | 3.48±0.09^#^ | 4.89±0.18**^##^ |
| LVAW, d（mm） | 0.76±0.02 | 0.75±0.03 | 0.78±0.03 | 1.01±0.04* | 0.84±0.02^#^ | 1.20±0.07**^#^ |
| LVPW, d（mm） | 0.70±0.02 | 0.71±0.04 | 0.73±0.03 | 0.93±0.03* | 0.76±0.04^#^ | 1.16±0.05**^##^ |
| LVID, d（mm） | 3.77±0.04 | 3.81±0.04 | 3.84±0.07 | 3.98±0.05 | 3.83±0.05 | 4.12±0.09* |
| FS（%） | 45.19±1.95 | 42.09±1.70 | 42.46±0.93 | 34.57±1.54** | 39.88±1.63^#^ | 26.36±1.49**^#^ |
| **Hemodynamic** |  |  |  |  |  |  |
| LVESP (mmHg) | 112.38±1.40 | 111.71±0.95 | 109.04±1.41 | 85.18±1.19** | 99.81±2.50*^#^ | 64.70±0.94***^##^ |
| LVEDP (mmHg) | 3.06±0.31 | 2.77±0.30 | 2.72±0.43 | 7.93±0.35** | 6.01±0.29*^#^ | 13.56±0.57***^##^ |

Values represent means ± SEs; n = 6 per group. **P*<0.05, ** *P*<0.01, *** *P*<0.001 versus Sham; ^#^*P*<0.05, ^##^*P*<0.01 versus WT of TAC. BW, body weight; HR, heart rate; LV mass, left ventricular mass; LV mass /BW, the ratio of LV mass to body weight; LVAW,d, LV anterior wall thickness at end-diastole; LVPW,d, LV posterior wall thickness at end-diastole; LVID,d, LV internal diameter at end-diastole; FS, fractional shortening; LVESP, left ventricular end systolic pressure; LVEDP, left ventricular end diastolic pressure.
