# Supplementary material for: Transmembrane tumor necrosis factor alpha attenuates pressure-overload cardiac hypertrophy via tumor necrosis factor receptor 2
Source: PLoS Biol. 2020 Dec 3;18(12):e3000967. doi: 10.1371/journal.pbio.3000967 (PMC7714153; doi:10.1371/journal.pbio.3000967)
Supplement: S2 Table — (DOCX) [file pbio.3000967.s008.docx]

S2 Table. Echocardiographic and hemodynamic analysis in mice injected with **rAAV-GFP, rAAV-TNFR1 shRNA** or **rAAV-TNFR2 shRNA** at 2 weeks after sham or TAC operation

|  | Sham | | | TAC | | |
| --- | --- | --- | --- | --- | --- | --- |
|  | Vector | TNFR1 | TNFR2 | Vector | TNFR1 | TNFR2 |
|  |  | shRNA | shRNA |  | shRNA | shRNA |
| BW（g） | 24.84±0.43 | 24.96±0.38 | 25.10±0.51 | 24.48±0.52 | 23.93±0.59 | 24.23±0.61 |
| HW/BW (mg/g) | 4.54±0.04 | 4.36±0.06 | 4.57±0.07 | 5.37±0.07* | 4.99±0.07*^#^ | 5.93±0.10**^#^ |
| **Echocardiography** |  |  |  |  |  |  |
| HR (b.p.m) | 460.3±15.7 | 450.2±14.7 | 457.2±15.4 | 446.8±21.2 | 429.5±18.5 | 437.0±20.7 |
| LV mass（mg） | 69.71±2.05 | 68.16±2.24 | 67.14±2.34 | 99.46±2.15 | 84.84±2.68 | 110.67±2.43 |
| LV mass/BW（mg/g） | 2.81±0.09 | 2.73±0.08 | 2.69±0.15 | 4.01±0.10** | 3.41±0.13*^##^ | 4.48±0.07**^#^ |
| LVAW,d (mm) | 0.75±0.03 | 0.73±0.02 | 0.77±0.02 | 0.98±0.02* | 0.87±0.01*^#^ | 1.17±0.02**^#^ |
| LVPW,d (mm) | 0.65±0.02 | 0.66±0.02 | 0.64±0.02 | 0.96±0.03* | 0.77±0.04^#^ | 1.19±0.02**^#^ |
| LVID, d（mm） | 3.81±0.07 | 3.78±0.05 | 3.74±0.07 | 3.95±0.05 | 3.72±0.05 | 4.04±0.07* |
| EF（%） | 66.31±1.30 | 65.52±2.05 | 66.19±1.60 | 57.39±2.25* | 65.85±1.54^#^ | 48.74±1.30**^#^ |
| FS（%） | 43.06±1.25 | 41.60±1.19 | 43.64±1.60 | 35.07±1.30** | 41.60±1.56^#^ | 27.35±1.88**^#^ |
| **Hemodynamic** |  |  |  |  |  |  |
| LVEDP (mmHg) | 2.45±0.42 | 2.77±0.32 | 3.14±0.39 | 9.61±0.82** | 6.28±0.47*^#^ | 15.46±1.39***^#^ |
| dP/dt_max_ (mmHg/s) | 10519±389 | 10389±341 | 10629±285 | 7211±197** | 7819±482** | 5425±401***^#^ |
| dP/dt_min_ (mmHg/s) | -9198±250 | -9024±311 | -9065±419 | -5325±155** | -6467±181*^#^ | -3479±178***^##^ |

Values represent means ± SEs; n = 6 per group. **P*<0.05, ** *P*<0.01, *** *P*<0.001 versus Sham; ^#^*P*<0.05, ^##^*P*<0.01 versus Vector of TAC. BW, body weight; HW/BW, the ratio of heart weight to body weight; HR, heart rate; LV mass, left ventricular mass; LV mass /BW, the ratio of LV mass to body weight; LVAW,d, LV anterior wall thickness at end-diastole; LVPW,d, LV posterior wall thickness at end-diastole; LVID,d, LV internal diameter at end-diastole; LVEDP, left ventricular end diastolic pressure; dP/dt_max_, peak instantaneous rate of left ventricular pressure increase; dP/dt_min_, peak instantaneous rate of left ventricular pressure increase decline.
