# Supplementary material for: Transmembrane tumor necrosis factor alpha attenuates pressure-overload cardiac hypertrophy via tumor necrosis factor receptor 2
Source: PLoS Biol. 2020 Dec 3;18(12):e3000967. doi: 10.1371/journal.pbio.3000967 (PMC7714153; doi:10.1371/journal.pbio.3000967)
Supplement: S3 Table — (DOCX) [file pbio.3000967.s009.docx]

**S3 Table. Echocardiographic and hemodynamic analysis in WT, TNFR1^-/-^ and TNFR2^-/-^ mice at 4 weeks after sham or TAC operation**

|  | Sham | | | TAC | | |
| --- | --- | --- | --- | --- | --- | --- |
|  | WT | TNFR1^-/-^ | TNFR2^-/-^ | WT | TNFR1^-/-^ | TNFR2^-/-^ |
| BW (g) | 25.48±0.55 | 25.29±0.45 | 25.18±0.58 | 25.42±0.49 | 25.17±0.64 | 24.32±0.77 |
| HW/BW | 4.36±0.10 | 4.30±0.13 | 4.27±0.11 | 5.54±0.11** | 4.92±0.12*^#^ | 6.23±0.22**^#^ |
| **Echocardiography** |  |  |  |  |  |  |
| HR (b.p.m) | 460.0±18.9 | 465.2±15.6 | 454.3±16.0 | 441.4±14.5 | 434.2±12.7 | 424.5±17.4 |
| LV mass（mg） | 76.98±2.71 | 75.11±3.56 | 77.57±2.92 | 114.91±3.87 | 98.23±3.84 | 127.78±5.15 |
| LV mass/BW（mg/g） | 3.02±0.08 | 2.97±0.12 | 3.08±0.08 | 4.46±0.14** | 3.90±0.09*^#^ | 5.27±0.21**^##^ |
| LVAW, d（mm） | 0.79±0.03 | 0.76±0.02 | 0.78±0.03 | 1.12±0.05** | 0.93±0.03*^##^ | 1.22±0.05** |
| LVPW, d（mm） | 0.70±0.02 | 0.68±0.03 | 0.72±0.02 | 1.06±0.06** | 0.87±0.03*^#^ | 1.14±0.07** |
| LVID, d（mm） | 3.85±0.04 | 3.80±0.05 | 3.79±0.04 | 4.14±0.03** | 3.89±0.09^#^ | 4.38±0.06**^#^ |
| EF（%） | 71.31±2.13 | 69.62±2.43 | 69.43±2.42 | 46.76±1.38** | 57.67±2.83*^#^ | 34.80±3.15**^#^ |
| FS（%） | 43.98±2.86 | 45.58±1.59 | 42.62±2.01 | 30.08±1.07** | 37.74±1.22*^#^ | 22.55±0.63**^#^ |
| **Hemodynamic** |  |  |  |  |  |  |
| LVEDP (mmHg) | 2.82±0.56 | 2.46±0.50 | 2.65±0.60 | 11.65±1.21*** | 7.56±0.75***^##^ | 17.39±0.69***^##^ |
| dP/dt_max_ (mmHg/s) | 10026±454 | 9707±367 | 9667±377 | 4945±145*** | 6784±275***^##^ | 2998±330***^##^ |
| dP/dt_min_ (mmHg/s) | 8331±541 | -7967±345 | -8038±510 | -4598±352*** | -6304±182*^#^ | -2709±221***^#^ |

Values represent means ± SEs; n = 6 per group. **P*<0.05, ** *P*<0.01, *** *P*<0.001 versus Sham; ^#^*P*<0.05, ^##^*P*<0.01 versus Vector of TAC. BW, body weight; HW/BW, the ratio of heart weight to body weight; HR, heart rate; LV mass, left ventricular mass; LV mass /BW, the ratio of LV mass to body weight; LVAW,d, LV anterior wall thickness at end-diastole; LVPW,d, LV posterior wall thickness at end-diastole; LVID,d, LV internal diameter at end-diastole; LVEDP, left ventricular end diastolic pressure; dP/dt_max_, peak instantaneous rate of left ventricular pressure increase; dP/dt_min_, peak instantaneous rate of left ventricular pressure increase decline.
