# Supplementary material for: Transmembrane tumor necrosis factor alpha attenuates pressure-overload cardiac hypertrophy via tumor necrosis factor receptor 2
Source: PLoS Biol. 2020 Dec 3;18(12):e3000967. doi: 10.1371/journal.pbio.3000967 (PMC7714153; doi:10.1371/journal.pbio.3000967)
Supplement: S4 Table — (DOCX) [file pbio.3000967.s010.docx]

**S4 Table. Antibodies used in this study**

|  | Primary antibodies | | |
| --- | --- | --- | --- |
| Antigen | Source | Species | Working dilution |
| TNFR1 | Santa Cruz | Mouse | 1:1000, WB |
| TNFR2 | Santa Cruz | Rabbit | 1:1000, WB |
| TNFR1 | ABclonal | Rabbit | 1:100, IFC; |
| TNFR2 | ABclonal | Rabbit | 1:100, IFC; |
| tmTNF-α | made in house | Rabbit | 1:100, IFC; 1:1000, WB |
| TACE | ProSci | Rabbit | 1:100, FACS |
| TACE | ABclonal | Rabbit | 1:100, IFC |
| Troponin T | abcam | Mouse | 1:200, IFC |
| p65 | CST | Rabbit | 1:1000, WB |
| p-p65 | CST | Rabbit | 1:500, WB |
| AKT | CST | Rabbit | 1:1000, WB |
| p-AKT | CST | Rabbit | 1:500, WB |
| GAPDH | Santa Cruz | Mouse | 1:1000, WB |
| TNF-α | LifeSpan BioSciences | Rabbit | 1:100, FACS |
|  | Secondary antibodies | | |
| anti-rabbit HRP | Jackson Biotech | Goat | 1:5000, WB |
| anti-mouse HRP | Jackson Biotech | Goat | 1:5000, WB |
| anti-rabbit FITC | Jackson Biotech | Goat | 1:100, FACS |
| anti-rabbit Cy3 | Servicebio | Goat | 1:100, IFC |
| anti-mouse FITC | Servicebio | Goat | 1:100, IFC |

WB：Western blot; IFC: Immunofluorescence; FACS: Flow cytometry
