# Supplementary material for: Transmembrane tumor necrosis factor alpha attenuates pressure-overload cardiac hypertrophy via tumor necrosis factor receptor 2
Source: PLoS Biol. 2020 Dec 3;18(12):e3000967. doi: 10.1371/journal.pbio.3000967 (PMC7714153; doi:10.1371/journal.pbio.3000967)
Supplement: S5 Table — (DOCX) [file pbio.3000967.s011.docx]

**S5 Table. Primer sequences for quantitative RT-PCR**

| Gene spiecies | | Primer sequence | |
| --- | --- | --- | --- |
| GAPDH | mice | F | TGTGTCCGTCGTGGATCTGA |
|  |  | R | TTGCTGTTGAAGTCGCAGGAG |
| BNP | mice | F | AGATAACCCCACCCCTACTCC |
|  |  | R | GTCTTCAACAACGGTGCCTCT |
| ANP | mice | F | CCTGTGTACAGTGCGGTGTC |
|  |  | R | AAGCTGTTGCAGCCTAGTCC |
| IL-6 | mice | F | CTGCAAGAGACTTCCATCCAGTT |
|  |  | R | GAAGTAGGGAAGGCCGTGG |
| IL-10 | mice | F | TTTGAATTCCCTGGGTGAGAA |
|  |  | R | GGAGAAATCGATGACAGCGC |
| IL-1β | mice | F | CAACCAACAAGTGATATTCTCCATG |
|  |  | R | GATCCACACTCTCCAGCTGCA |
| TNF-α | mice | F | GCCTATGTCTCAGCCTCTTCTCA |
|  |  | R | ACTCCAGCTGCTCCTCCACTT |
| ANP | Rat | F | CCTCTTCCTGGCCTTTTGG |
|  |  | R | TGTGTTGGACACCGCACT |
| BNP | Rat | F | TCTGCTCCTGCTTTTCCTTA |
|  |  | R | GAACTATGTGCCATCTTGGA |
| IL-6 | Rat | F | GGATATAACCAGGAAATTTGCC |
|  |  | R | GTATTGCTCTGAATGACTCTGG |
| IL-1β | Rat | F | CTACCTATGTCTTGCCCGTGG |
|  |  | R | GGATTTTGTCGTTGCTTGTCTC |
| IL-10 | Rat | F | ACTGCTATGTTGCCTGCTCTT |
|  |  | R | AATGCTCCTTGATTTCTGGG |
| TNF-α | Rat | F | TGTGCCTCAGCCTCTTCTCATT |
|  |  | R | TTGTCACTCGAGTTTTGAGAAGATG |
| TNFR1 | Rat | F | TGTGTAACTGCCACGCAGGA |
|  |  | R | AAGATAACCAGAGGCAACAGCAC |
| TNFR2 | Rat | F | GCGAACTGCTTCATCCTGGT |
|  |  | R | GTGCTGTGGTCAATAGGTGCTG |
| TACE | Rat | F | CTCTCTTTATCTAATATCCAGCAGCAC |
|  |  | R | ACCACGACTCGCAAGTTCTGT |
| GAPDH | Rat | F | CAGTGCCAGCCTCGTCTCAT |
|  |  | R | AGGGGCCATCCACAGTCTTC |

F: forward; R: reverse
