# Supplementary material for: Transmembrane tumor necrosis factor alpha attenuates pressure-overload cardiac hypertrophy via tumor necrosis factor receptor 2
Source: PLoS Biol. 2020 Dec 3;18(12):e3000967. doi: 10.1371/journal.pbio.3000967 (PMC7714153; doi:10.1371/journal.pbio.3000967)

# Raw images of western blotting

## Related to Fig 2A

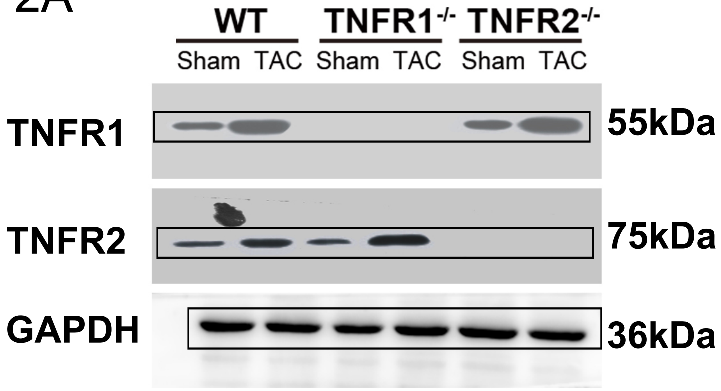


## Related to Fig 2C

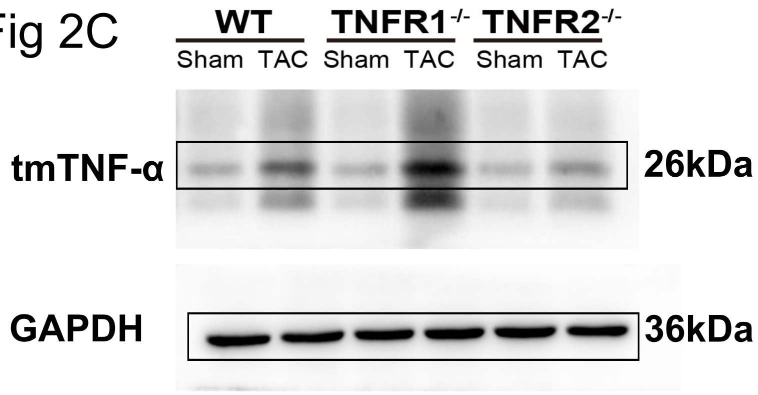


## Related to Fig 2H

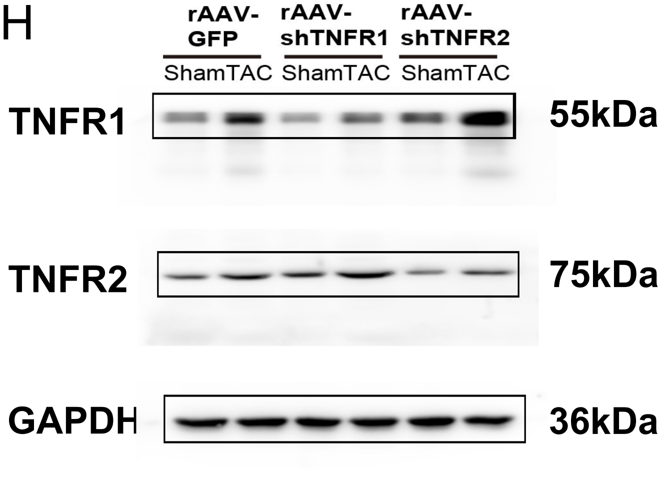


## Related to Fig 2K

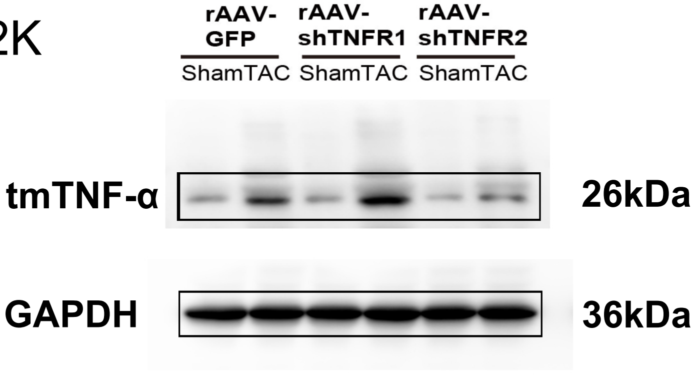

Related to Fig 4C

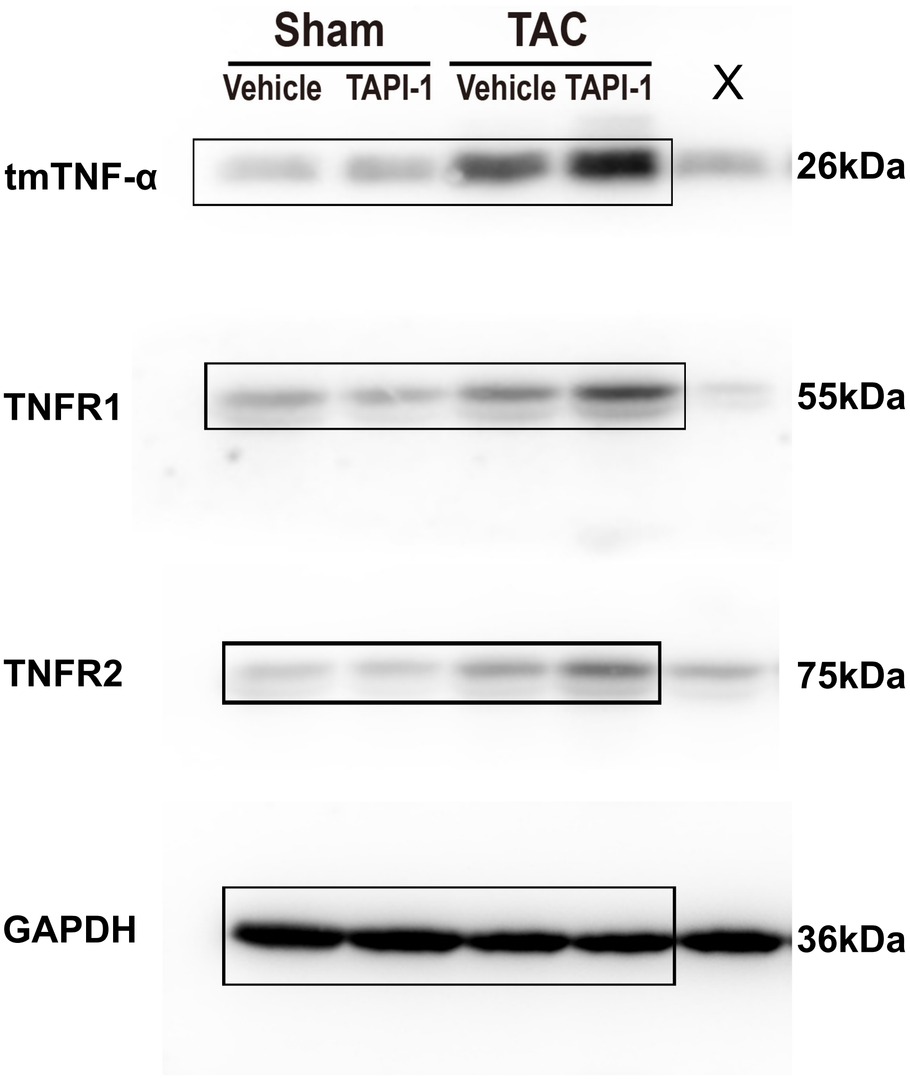

## Related to FIG 7A

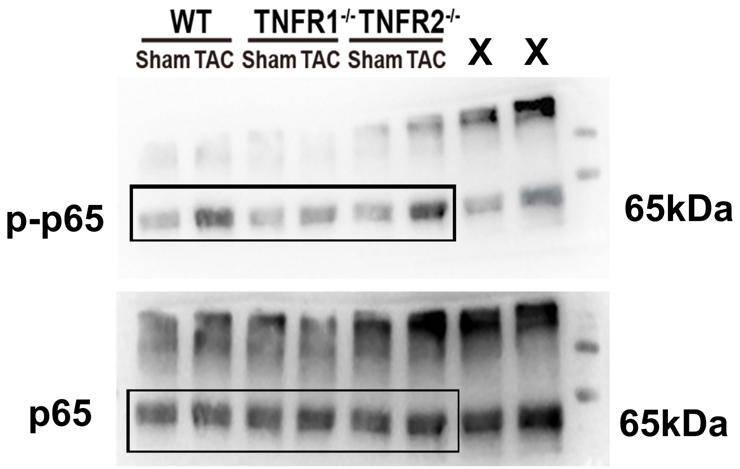


## Related to FIG 7B

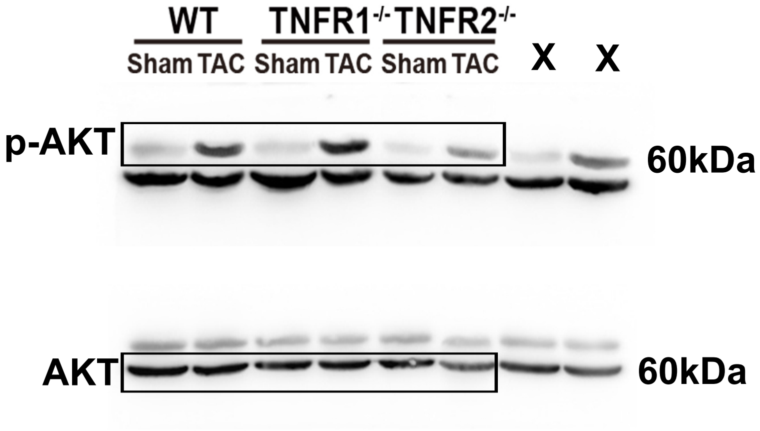


## Related to FIG 7C

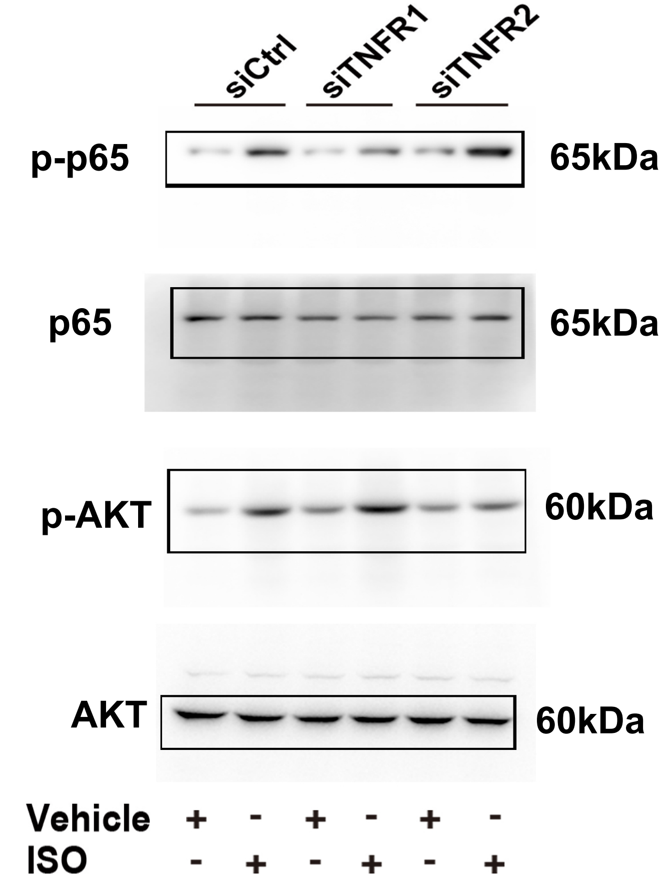


## Related to FIG 7D

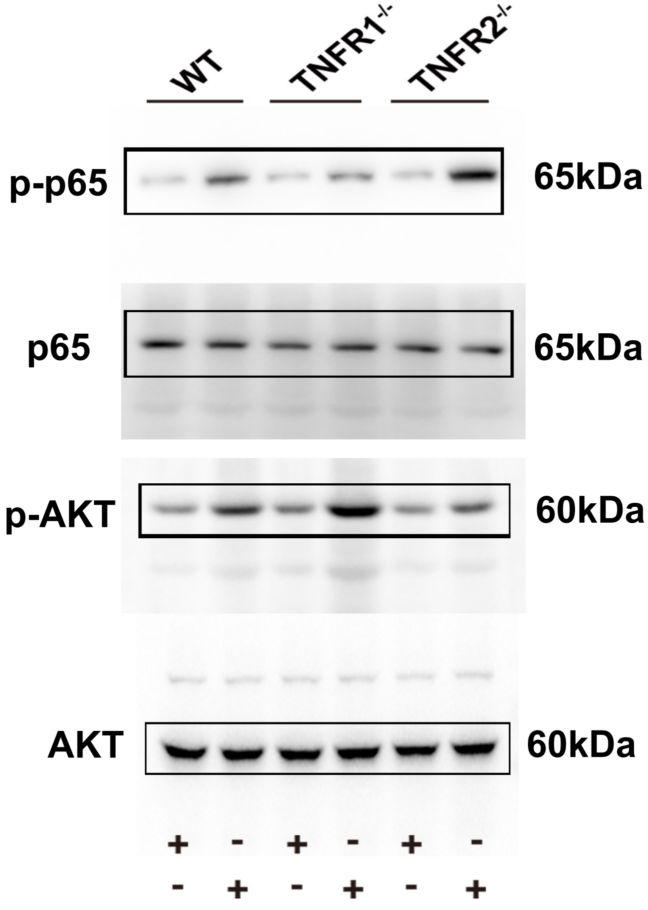


## Related to FIG 7E

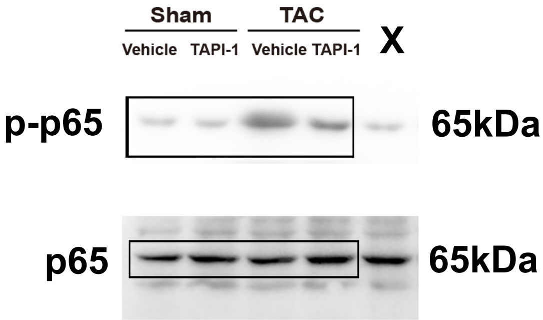


## Related to FIG 7F

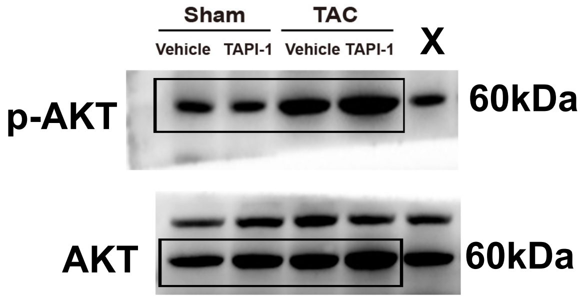

Related to FIG 7G

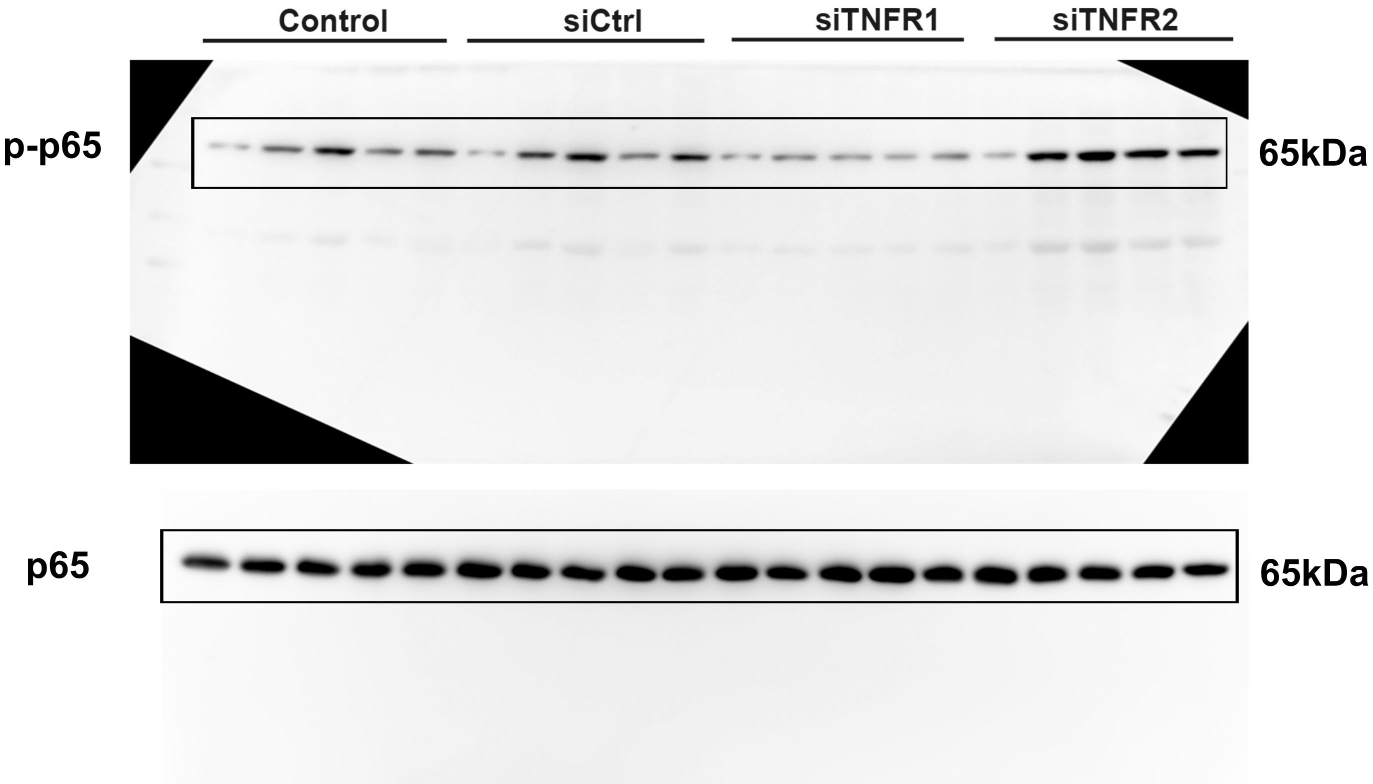


Related to FIG 7H

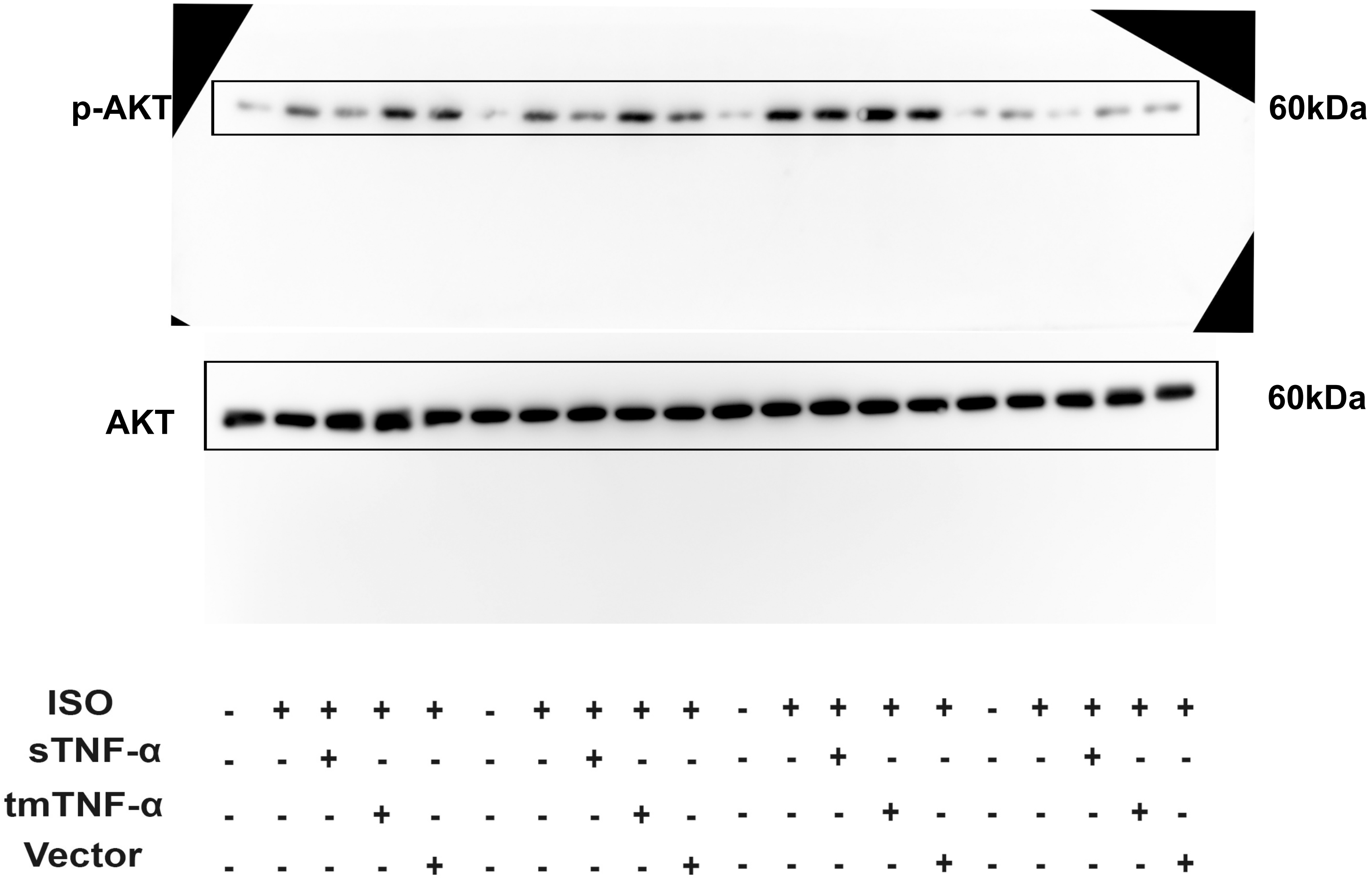

Related to FIG 7K

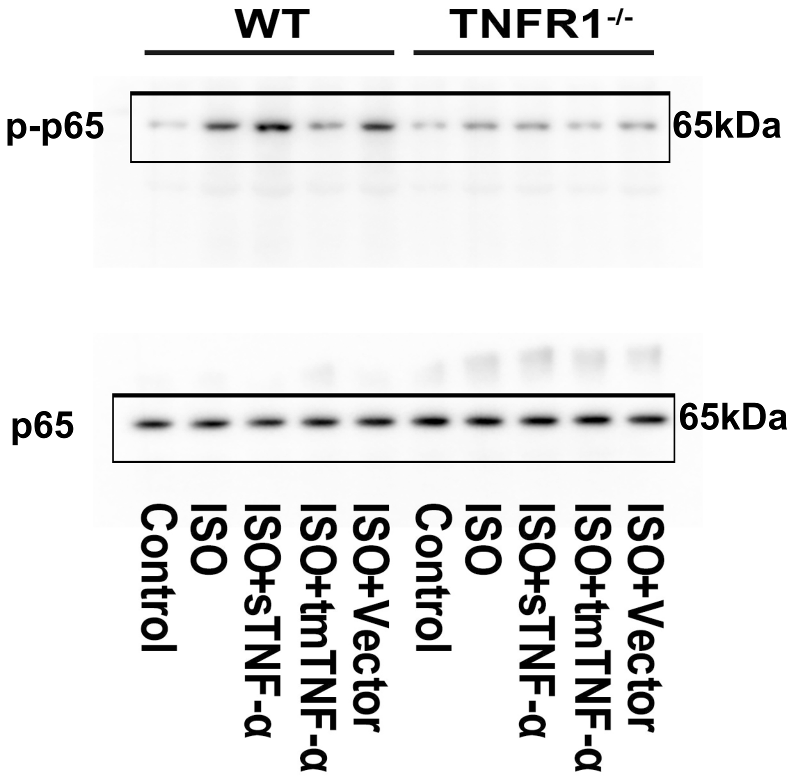


Related to FIG 7L

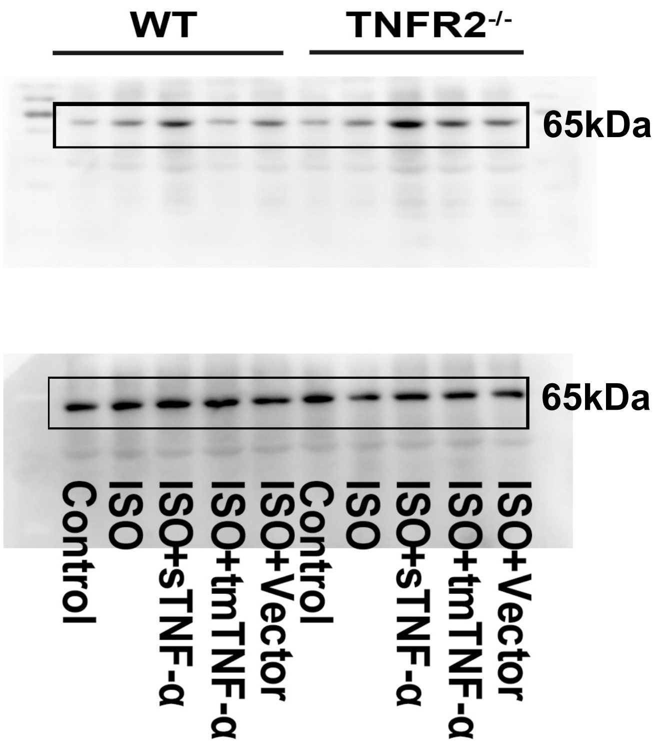


Related to FIG 7M

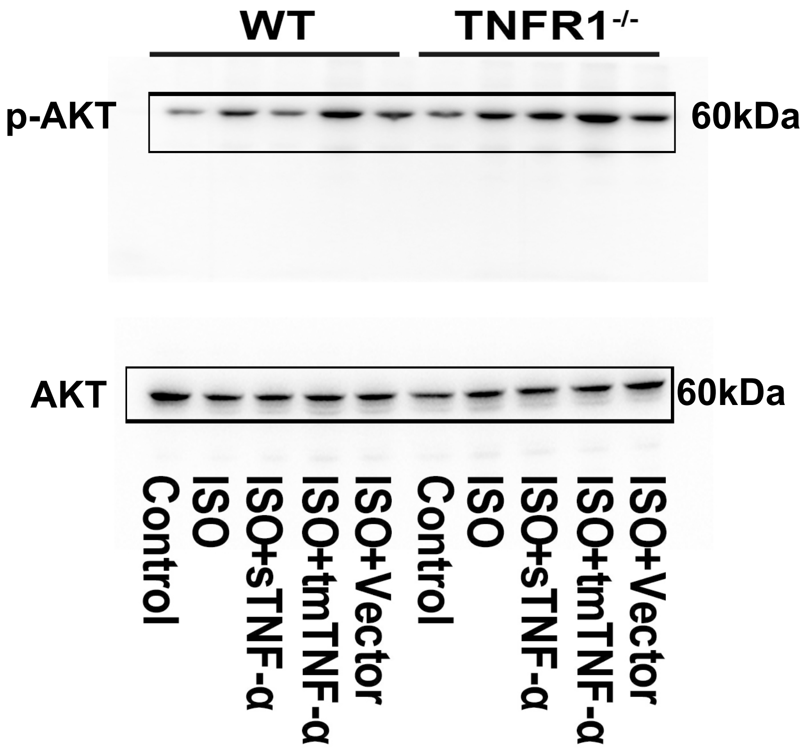


Related to FIG 7N

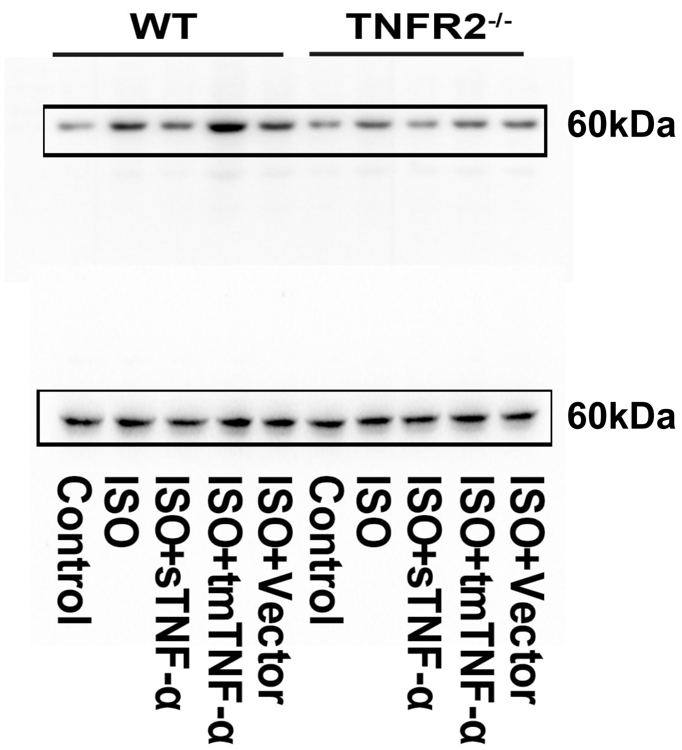

## Related to Fig S2E

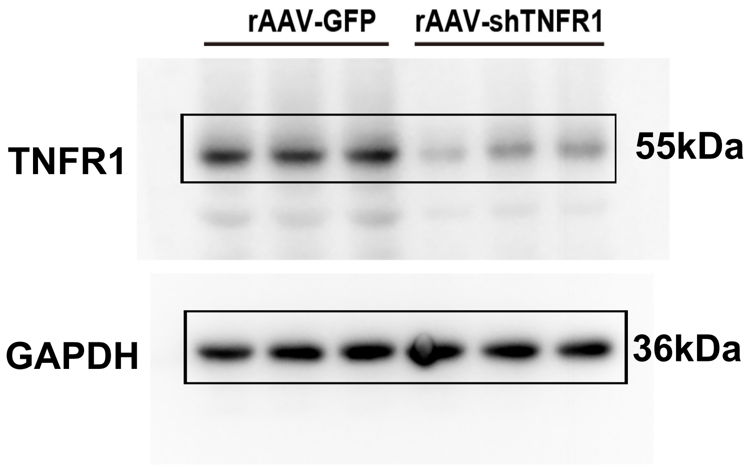


## Related to Fig S2F

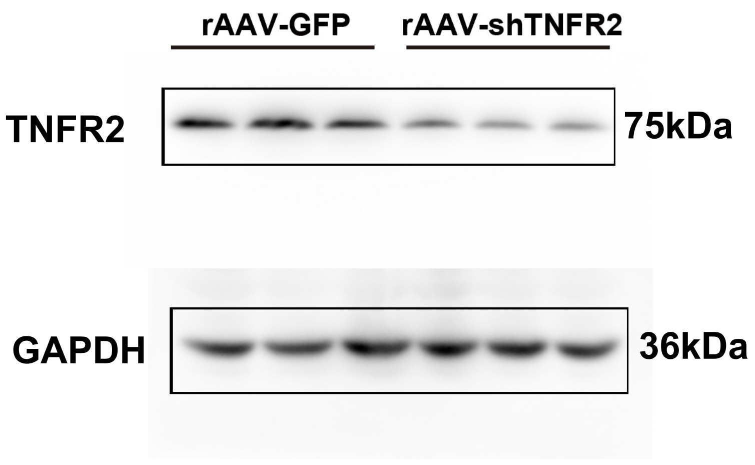


## Related to Fig S3I

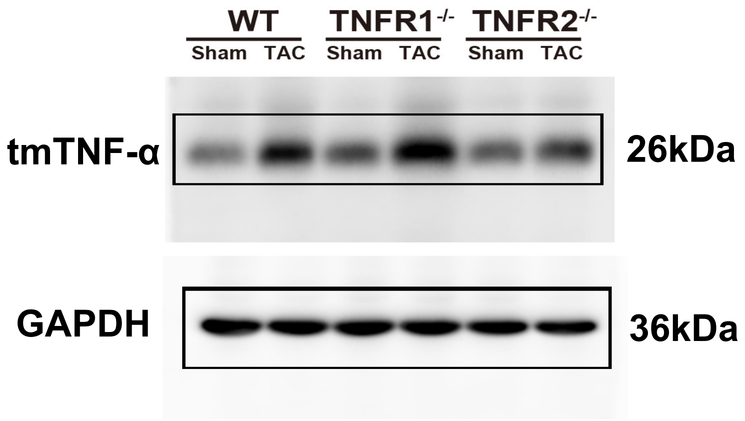


## Related to Fig S4B

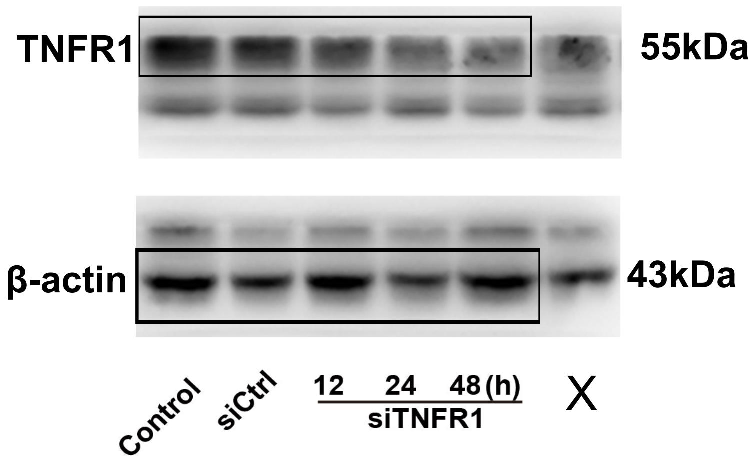


## Related to Fig S4D

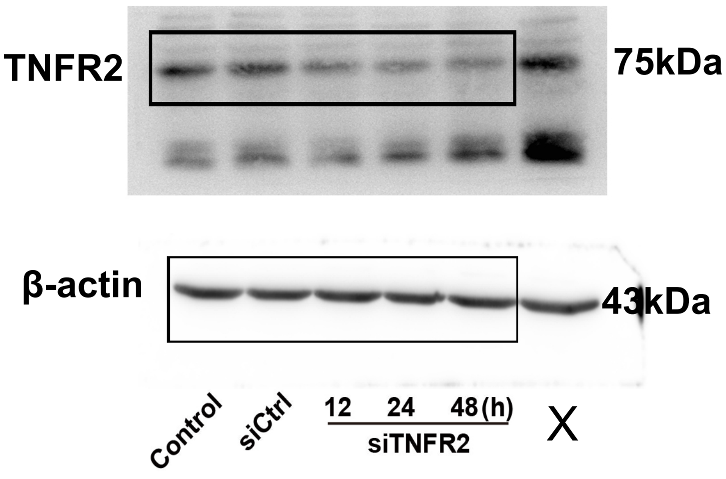

Supplement: S1 Raw Images — (PDF) [file pbio.3000967.s013.pdf]
